# Molecular Dynamics and Machine Learning Give Insights on the Flexibility–Activity Relationships in Tyrosine Kinome

**DOI:** 10.1021/acs.jcim.3c00738

**Published:** 2023-07-18

**Authors:** Sarmistha Majumdar, Francesco Di Palma, Francesca Spyrakis, Sergio Decherchi, Andrea Cavalli

**Affiliations:** † Computational & Chemical Biology, Fondazione Istituto Italiano di Tecnologia, Via Morego 30, I-16163 Genova, Italy; ‡ Department of Drug Science and Technology, University of Turin, via Giuria 9, I-10125 Turin, Italy; § Data Science and Computation, Fondazione Istituto Italiano di Tecnologia, Via Morego 30, I-16163 Genova, Italy; ∥ Department of Pharmacy and Biotechnology, University of Bologna, Via Belmeloro 6, I-40126 Bologna, Italy

## Abstract

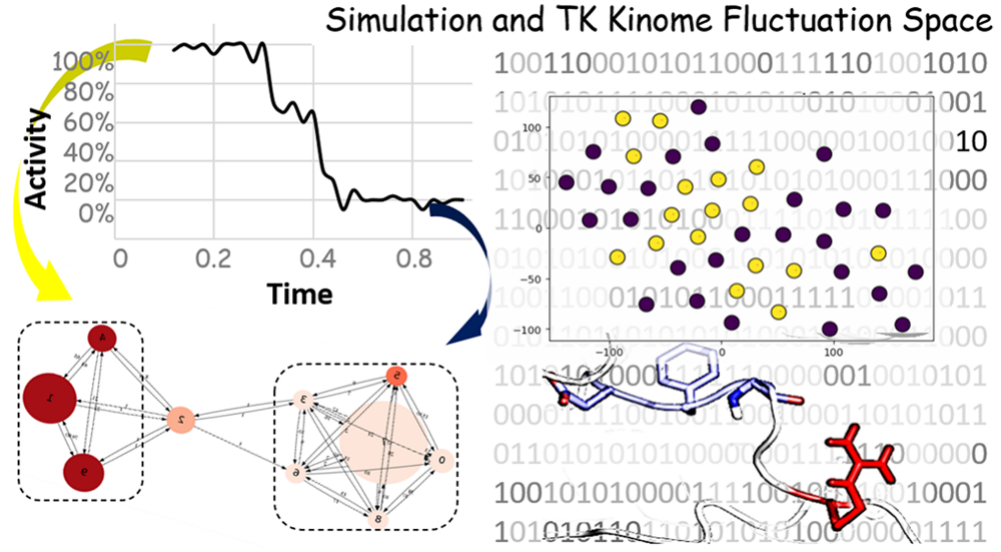

Tyrosine kinases are a subfamily of kinases with critical roles in cellular machinery. Dysregulation of their active or inactive forms is associated with diseases like cancer. This study aimed to holistically understand their flexibility–activity relationships, focusing on pockets and fluctuations. We studied 43 different tyrosine kinases by collecting 120 μs of molecular dynamics simulations, pocket and residue fluctuation analysis, and a complementary machine learning approach. We found that the inactive forms often have increased flexibility, particularly at the DFG motif level. Noteworthy, thanks to these long simulations combined with a decision tree, we identified a semiquantitative fluctuation threshold of the DGF+3 residue over which the kinase has a higher probability to be in the inactive form.

## Introduction

Tyrosine kinases (TKs), both receptor and nonreceptor, are a large and diverse family of proteins found in unicellular and multicellular organisms across all holozoans.^1,2^ TKs control and regulate several biological processes, including cell-to-cell communication, cell growth, motility, differentiation, metabolism, and cell apoptosis.^3^ TKs frequently transmit signals related to these processes by modulating signal transduction via phosphorylation of tyrosine residues (i.e., the transfer of an ATP phosphate to a tyrosine side chain on protein substrates). In humans, dysregulated TKs participate in the development of many diseases, including neoplasms, diabetes, and developmental congenital syndromes.^4^ TKs form a class of oncogenes involved in most forms of human cancer.^5,6^ These kinases (e.g., EGFR-TK, ABL1, JAK2) are key players in pathways inducing many neoplastic changes (e.g., malignant transformation, growth, metastasis) and are preferentially mutated in tumor cells.^7−9^

**Figure 1:**
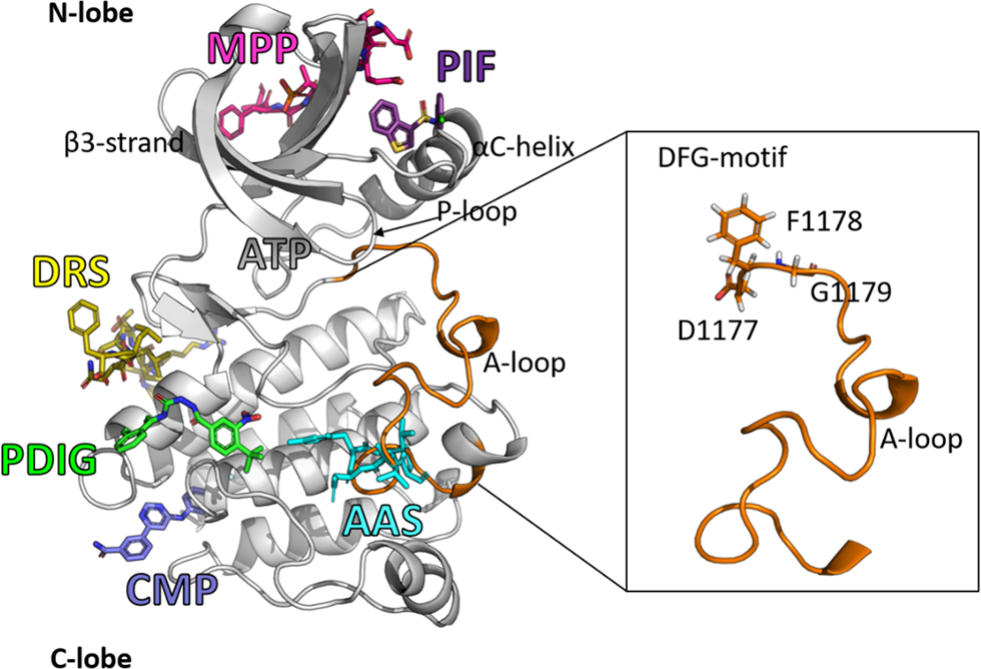
Map of the analyzed pockets and key activity regions of a Tyrosine Kinase. In the inset, the A-loop (orange) with the side chains of the DFG motif is shown in sticks (residue numbering according to IRK, PDB ID 5hhw(33)).

Over the past three decades, high-resolution structural studies have provided the molecular basis for understanding the mechanisms by which TKs are regulated and, in turn, regulate downstream processes. A kinase’s activation state is determined by several structural features, which are mostly found in the activation loop (A-loop) and the αC-helix^10−12^ (Figure 1 shows key TK regions). Kinases can adopt a closed or open conformation of the A-loop and a stretched or collapsed P-loop (the “phosphate-binding loop” or “glycine-rich” loop,) which are potential hallmarks of the open/closed state of the active site (e.g., c-MET, ABL1).^11,13^ The kinase domain’s “open state” facilitates the active conformation, and the “closed state” favors an inactive conformation. TK features are often associated with activity.^14^ These features include: (i) the opening of the A-loop to an extended state and an inward rotation of the αC-helix resulting in the formation of the typical K/E salt bridge; (ii) the rotation of the αC-helix altering the hydrophobic regulatory spine (R-spine); (iii) the formation of a tight electrostatic network from the C-lobe catalytic loop to the N-lobe αC and β-sheet, and across the A-loop, which comprises six polar highly conserved residues in the catalytic domain acting as a switch during activation; and (iv) the presence of charge asymmetry in the A-loop. Phosphorylation of the A-loop is also crucial to most TK activation because it rigidifies the structure, upregulating the kinase activity (e.g., Y416 in the Src family TKs,^14^ Y1007/Y1008 in the Janus kinase 2^15^). Hence, conformational plasticity is necessary for TK activity.^16^ Several in silico studies of TKs have described the mechanisms of kinase activity switching (e.g., KIT,^17^ c-Src,^18,19^ EGFR,^20^ FGFR2^21^). In 1996,^22^ researchers attempted to summarize the structural basis for kinase regulation in order to rationalize the activation segment’s role, based on the distinction between active and inactive kinases. More recently, researchers have proposed several methods to distinguish active from inactive forms. These include: (i) approaches based on DFG-in/DFG-out conformation along with the αC-helix orientation (in/out/intermediate);^23,24^ (ii) knowledge based kinase-ligand interaction space determination;^25,26^ (iii) Brooijman’s method;^27^ (iv) ABC method;^28^ (v) hydrophobic R-spine;^29,30^ and (vi) normal-mode analysis.^31^

The present study used an unbiased big-data-driven protocol to identify regularities and differences in TKs and, thus, understand their activity. We conducted a massive simulation campaign of about 120 μs coupled with analyses based on machine learning (ML) methods. While simulating a single complex can generate a wealth of highly specific information,^32,33^ we took a more holistic approach based on high-performance computing and ML. Indeed, a single-frame or single-protein analysis would not have produced the present findings. In detail, starting from the produced trajectories, we performed a TK-kinome-wide analysis of the time-averaged volumes of pockets and the probability distributions of pockets connections. Then, we characterized each TK’s activity profile with a dynamical quantity i.e., fluctuations in residue backbones via root-mean-square fluctuations (RMSF). Finally, we focused on three kinases with interesting predicted dynamical activity/inactivity patterns: the insulin receptor kinase (IRK), vascular endothelial growth factor receptor 2 (VEGFR2), and Bruton’s tyrosine kinase (BTK).

## Results and Discussion

We simulated 43 TKs for a total sampling time of about 120 μs. Each system was simulated for at least 1 μs, with 3 μs being the typical sampling time. We employed simple plain MD as it already proved reliable for studying pockets cross-talks,^33^ and we were not aiming to the detection of activity switching, which we already studied elsewhere.^32^Table S1 reports details of the individual systems, their PDB IDs, and the activity/inactivity information from each structure’s reference paper. Where the reference paper did not clearly indicate the active/inactive state, we used Kinconform^30^ to infer the initial state (see Methods for details). Where the active/inactive state was clearly indicated in the literature, our predictions fully agreed with these indications.

We assigned a code to each of the 43 molecular dynamics (MD) simulations in the form of NAME[α]-β_[i/a]_ (see Table S1), where NAME is the abbreviated TK name, α is the optionally indicated TK isoform, β is the index of the simulation (where multiple PDBs of the same TK were simulated), and a subscripted “i” or “a” indicates structures labeled ab initio as inactive or active, respectively. Hereafter, we use the sequence numbering of the insulin receptor kinase (IRK, PDB ID 5hhw)^34^ as reference, unless otherwise specified.

### Pockets in the TK Kinome

**Figure 2:**
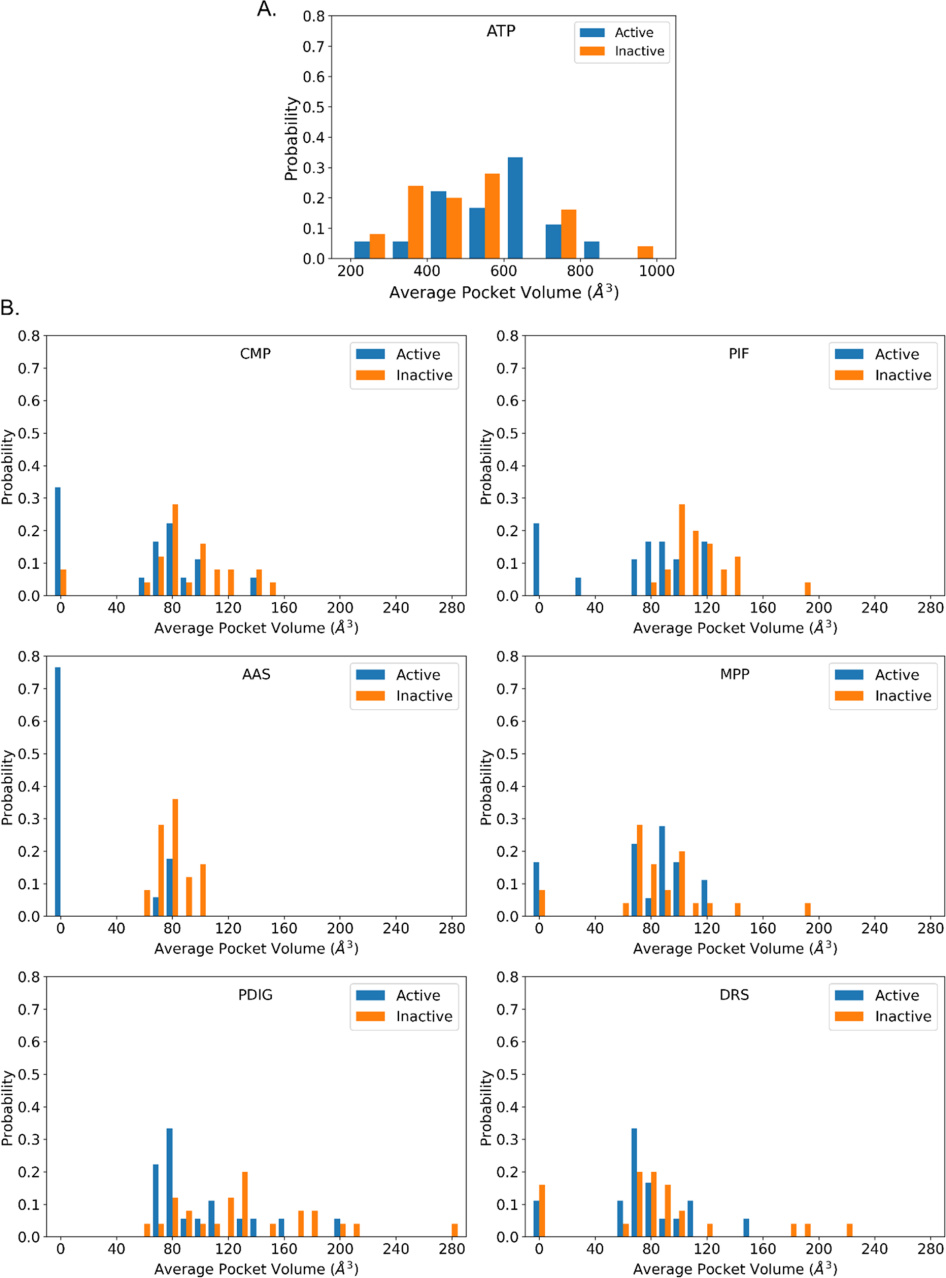
Distribution of the average volumes for all the analyzed pockets divided into active (blue) and inactive (orange) kinases; A. ATP pocket, and B. Allosteric sites. A volume of zero means that the corresponding pocket was not detected during the simulation in at least one system.

We statistically analyzed the dynamical behavior of pockets (i.e., from trajectories) in the TK kinome to understand the difference between pockets in active and inactive TKs. We analyzed the ATP binding site and the six pockets indicated in Figure 1 (AAS, CMP, DRS, PDIG, PIF, MPP), which are a subset of the 12 alternative sites whose “ligandability” was tested by Yueh et al.^35^ The other six pockets were excluded. In detail, DEF (typical of the MAPK family)^36^ was excluded because the folds of MAPKs and TK at DEF are not comparable. MT3 and DFG are very close to the ATP site, with which they often merge. EDI (i.e., EGFR-family Dimerization Interface) is at the interface between two kinases, so found only upon dimerization.^37^ PMP and LBP are absent from the TK family.^35^ Upon simulating the TKs via plain (unbiased) MD, we ran Pocketron^33^ to estimate each pocket’s time-averaged volume and characterize the interpocket communication network (see Methods for details). This shed light on how activity/inactivity correlates with the distribution of pocket volumes and pocket connectivity. The existence of a link between two pockets indicates a degree of flexibility in neighboring residues but might also indicate an allosteric communication between the two pockets. Here, we are interested in how the number of links is statistically distributed.^33^ Collectively, these statistics provide information about the TK family and offer a global dynamical data-driven vision of the TK “pocketome”. In Figure 2, we report the statistics (histogram) of the time-averaged volumes for each kinase pocket according to the active-inactive state of the TKs. Notably, the ATP site volume is almost independent of the active/inactive state. The two histograms (orange for inactive, blue for active, Figure 2A) are significantly superimposed, with an average volume of about 500–600 Å^3^, as confirmed by the two-means Welch’s *t* test^38^ (significance threshold = 0.05, *p* = 0.174, see Methods for further details). The inactive state has a marginal propensity to acquire bigger volumes. For the other pockets (Figure 2B), the active or inactive TK state marks a difference, which may be more or less noticeable depending on the specific case. In general, the pocket volume distribution is broader for inactive TKs than for active ones. This is consistent with the intuitive expectation that there are many ways to be inactive, while the requirements for activity are stricter and, thus, less variable. In mechanistic terms, pockets from inactive TKs, which are possible allosteric sites, are less rigid and tend to adopt different shapes and sizes. Hence, we observed a left-shift of the distribution for active TKs, i.e., they generally have smaller volumes.

Moreover, the probability of a null volume (pocket absence) is much higher for active than for inactive kinases. These pockets, in active TKs forms, tend to be more elusive. This is particularly relevant for the AAS pocket, which is absent from 75% of active kinases but always present for inactive kinases, albeit with a relatively small volume. To quantitatively and rigorously confirm this qualitative evidence (Figure 2B), we performed the statistical two-means Welch’s *t* test on the active vs inactive TKs pockets; we checked if there was statistically significant evidence of difference between volumes in active and inactive forms. For the CMP, PIF, AAS, and PDIG pockets, a difference was indicated by the *p*-values (0.024, 7.472e-04, 2.351e-07, and 0.022, respectively). However, the differences in the mean volumes of the two populations for DRS and MPP were not statistically significant (0.432 and 0.426, respectively).

**Figure 3:**
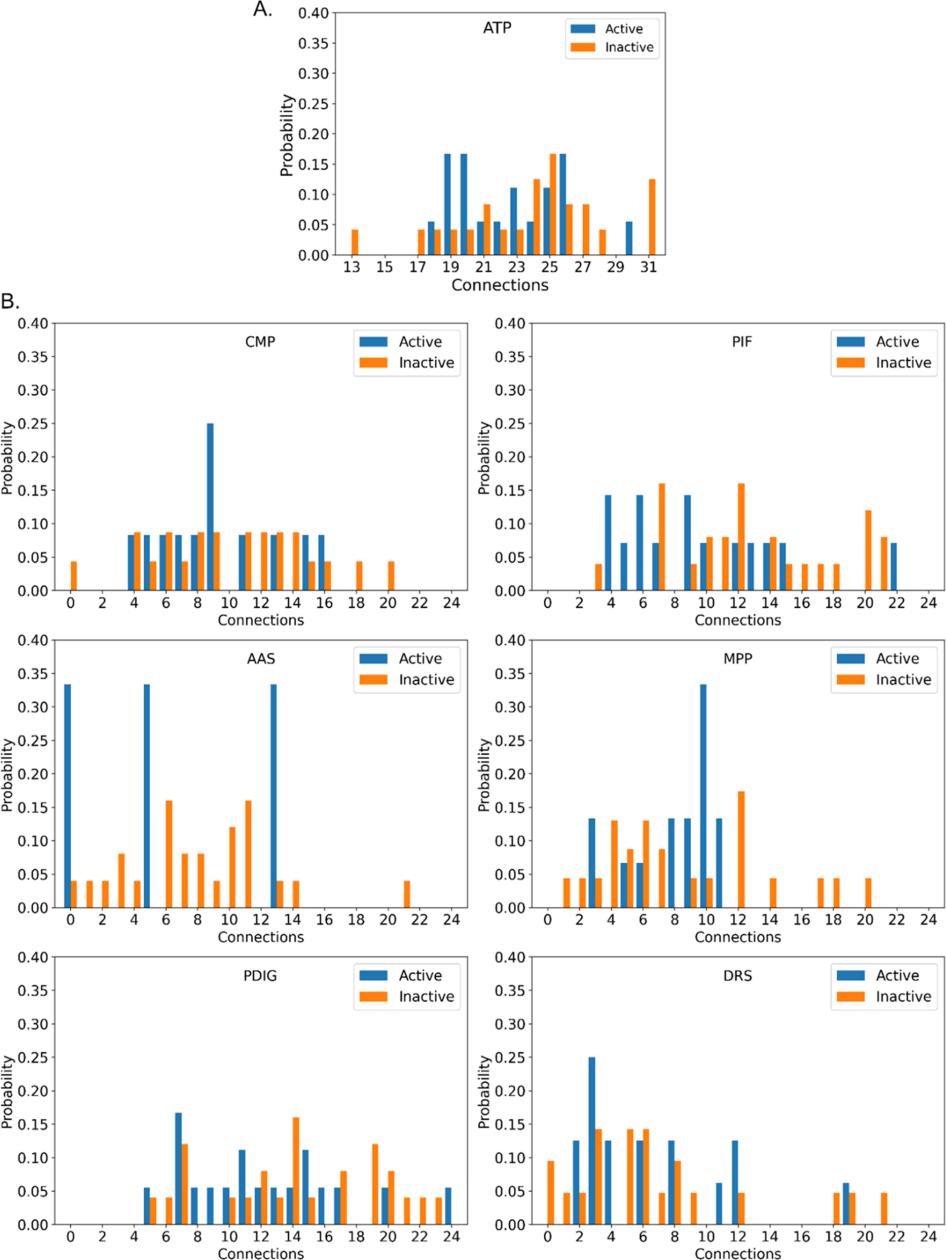
Distribution of the connections for all of the analyzed pockets divided into active (blue) and inactive (orange) kinases. A. ATP pocket, B. Allosteric sites.

In the second analysis, we collected statistics on each pocket’s connections, thus evaluating their ability to establish a network around themselves (Figure 3). This is a direct measure of flexibility and may indicate the propensity to create allosteric communication. Here, the active and inactive forms were slightly more homogeneous (Welch’s *t* test *p*-values: ATP 0.460, CMP 0.560, DRS 0.833, AAS 0.666, PDIG 0.207). For PIF and MPP, the distributions showed a pronounced left-shift (*p* = 0.086 and 0.061, respectively), which was only slightly above the significance threshold. Together with the previous observation, this shows that pockets in active TKs are rarer and slightly less connected to each other. On average, the TKs in active forms are more stable and less flexible. Interestingly, this result was obtained via plain MD simulations only, avoiding the potential unphysical bias associated with enhanced sampling.

Plasticity is a widely studied topic in kinases. Other researchers have also reported on flexibility patterns. Chen et al.^39^ reached similar conclusions using a kinematic flexibility analysis. Kornev et al.^29^ found that the unconstrained magnesium-binding loop (i.e., the part of the activation segment that includes the DFG motif and the two following residues) becomes flexible and can attain different inactive configurations. Levinson et al.^40^ and Vogtherr et al.^41^ reported this observation for ABL and p38 kinases, respectively, in which the DFG motif flips between in and out conformations in the inactive state. This confirms that active kinases, in particular, the activation segment (from DFG to DFG+6), are generally less flexible than inactive kinases. Hence, flexibility and fluctuations may help to identify the state of a kinase. Below, we use ML to investigate this point in greater detail.

### Fluctuations and Activity

To explicitly relate activity and fluctuations, we created an ad hoc data set of root-mean-square fluctuation (RMSF) values, where columns and rows represent residues and kinases, respectively, aligned according to sequence similarity (Figure S1). Each kinase was labeled as active or inactive according to its average activity state during the MD trajectory (Figure S2). The activity estimation for each frame was obtained with Kinconform^30^ and it was fully consistent with data coming from crystals and literature when the data were available.

**Figure 4:**
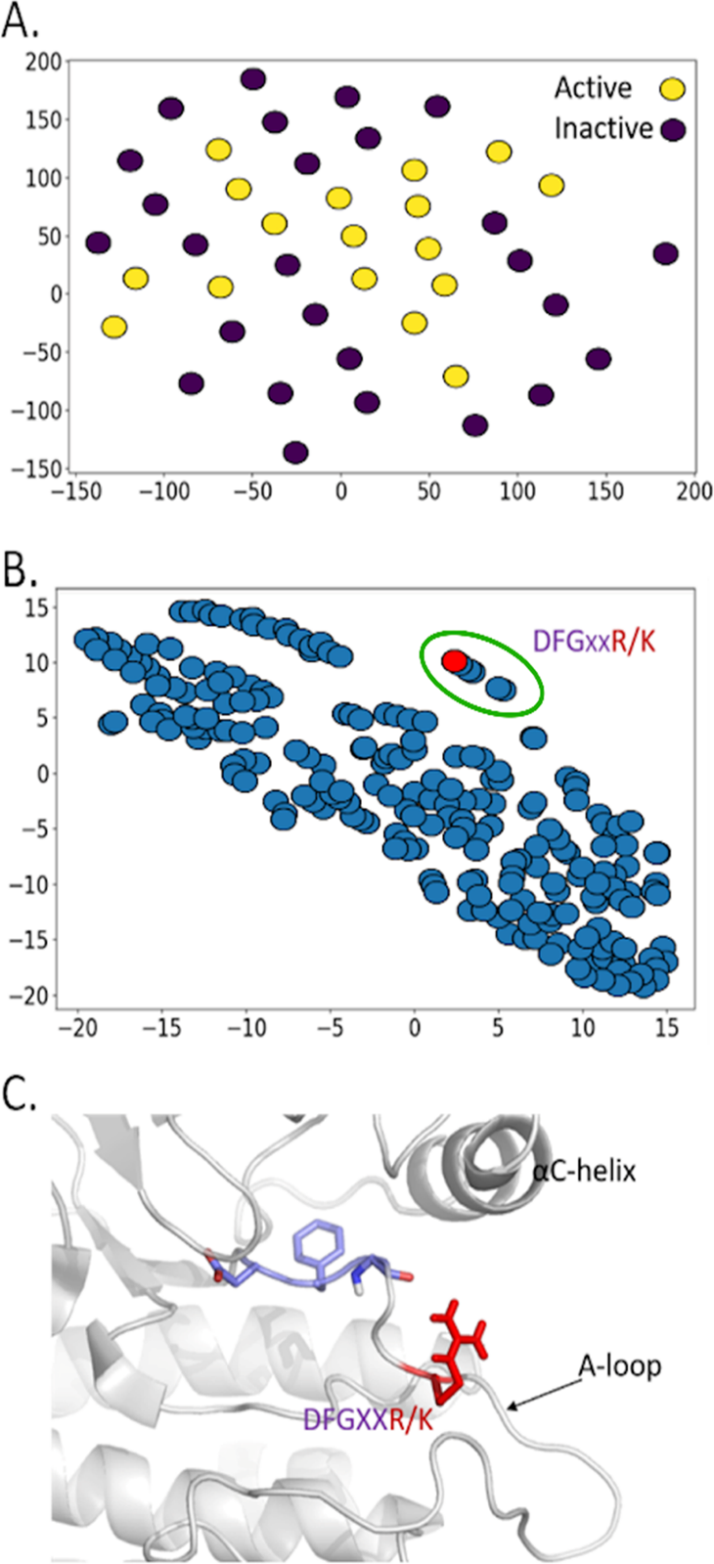
A. t-SNE projection of active and inactive TK proteins. B. t-SNE projection of the transposed space, with nearby residues having similar fluctuations. The red dotted line represents the DFG+3 residue, which determines the tree decision rule. The blue dots inside the green ellipses show the residues with fluctuations correlated to those of DFG+3. C. The DFG+3 arginine residue is colored red; in some TKs, a lysine replaced the arginine.

First, the matrix was projected into a 2D space with the t-SNE algorithm^42,43^ (Figure 4A). Inactive kinases were slightly more scattered than active ones, yet active kinases failed to create a well-defined single cluster. Next, to visualize the fluctuations, the transposed data set matrix was projected with the t-SNE^42,43^ (Figure 4B). Nearby residues showed similar fluctuations. The plot shows that residue fluctuations are often correlated (points can be clustered).

To understand if any relation holds between fluctuation and activity, we built a classifier (a decision tree) using the fluctuations as input and activity/inactivity as a prediction target. To make the tree as interpretable as possible and avoid overfitting, we constrained it to contain only one if-then-else rule.^44^ Data were randomly split into 30 training samples and 13 validation samples. We predicted activity with a balanced accuracy (see Methods) of 72.96% ± 11.78% (one standard deviation) by randomizing the splits 100 times while keeping the same sample ratio (0.7). As the tree depth is one, the final model is just a single rule, with a threshold on the fluctuation of one single residue. The trained model systematically identified a highly conserved lysine (or arginine) located at the DFG+3 position, namely in the activation segment (residue 1182 in our reference structure),^34^ the juxtaposed residue after the magnesium-binding loop. This is consistent with the role of DFG+3 in activation.^29^ However, to the best of our knowledge, there are no reports in the literature of a specific fluctuation threshold. We found that, if the fluctuation is below a threshold of 0.9 ± 0.2 Å, the kinase is classified as active; otherwise, it is inactive. This was quantitatively confirmed with the Lasso method,^45^ which also identified this residue. For corroboration, we performed an ablation study of the key K/R residue. We removed this residue from the matrix and checked which residue was selected from the tree. After ablation, the decision tree predicted the DFG+2 residue to be the best predictive residue for the kinase activity. Indeed, in Figure 4B, the DFG+2 and DFG+3 residues show highly correlated fluctuations. Removing the DFG+2 residue led to the identification of the DFG+1 residue. Removing the DFG+1 residue caused a significant decrease in the classification accuracy. For all of these cases, the rule consistently predicts activity when the fluctuation threshold is not exceeded. This indicates the overall importance of the movement of the A-loop backbone and points to the activation segment as a dynamically well-characterized region for determining the TK activity. In summary, a modest fluctuation of the loop may indicate activity, whereas a greater fluctuation tends to indicate an inactive state. Further checks were done on the possible bias induced by loop-reconstruction, as we modeled the activation loop in 13 out of 43 systems, and out of 13 only on 4 systems was the DFG+3 residue involved. First, we removed the 4 systems where the DFG+3 residue was reconstructed; we found a balanced accuracy of 68.51% ± 11.55%. Next, we removed all 13 kinases for which a loop reconstruction has been done, this time finding 65.64% ± 12.31%. To understand better if the 13 loop-reconstructed-kinases might represent a source of bias, we built a decision tree with these kinases only; we found in this case a balanced accuracy of 82.99% ± 23.53%. As in the 13 systems, 9 were inactive; taken together, all these data might suggest that loop reconstruction could contribute as a source of bias if we hypothesize that it affects more the behavior of inactive kinases (rendering them more flexible than expected). Nevertheless, we still got a more than chance result, even excluding all loop-reconstructed structures.

Given this fluctuation pattern at DFG+3, we characterized it further by analyzing the interactions of this residue in the 43 simulations. Figure S4 reports the progress of the interactions established during the trajectory of a pair of TKs. There is a clear difference between the inactive and active C-terminal TK domains of Janus kinase 2: JAK2-1_i_ (PDB ID 3ugc)^46^ and JAK2-2_a_ (PDB ID 6bbv)^47^ respectively. They were chosen as a representative case of the emerging picture, clearly showing the different trends for active and inactive TKs (irrespective of the presence of a K or R at the DFG+3 position).

The interactions established by DFG+3 in the active TK were stable during the simulation (Figure S4, panel B). However, inactive TK interactions were clearly fluctuating and, thus, unstable and nonspecific (Figure S4, panel A). For simulations without a stable activity plot (Figure S2), the DFG+3 interaction analysis found a mixture of stable and unstable interactions (e.g., BTK, Figure S4, panel C). Overall, the DFG+3 residue appears stable for active kinases, but the stabilizing partner residue depends on the specific TK.

In a nutshell, inactive kinases are more flexible both globally and locally. Flexibility thus tends to indicate activity status. The most relevant residue is DFG+3 (not DFG itself), whose flexibility/fluctuation can be considered a new semiquantitative hallmark of activity/inactivity. Interestingly, this residue is conserved (i.e., R or K) in all 43 TKs considered in this work and is highly conserved in the TK family. For the full set of TKs considered by Modi and Dunbrack,^48^ 85 of the 94 structures bear a positively charged arginine, lysine, or histidine at DFG+3. Moreover, DFG+3 is a positively charged residue in 280 of the 497 protein kinase domains of the full human kinome.^48^

### System Specific Analysis

In this section, we analyze in depth some systems that apparently switch activity (as predicted by Kinconform^30^). While full activation/inactivation transition requires time scales beyond our scope, our simulations still point to relevant conformational changes. We carried out this analysis for VEGFR2, IRK, and BTK, taking advantage of both the activity predictor and cluster analysis.

### Vascular Endothelial Growth Factor Receptor 2 (VEGFR2)

**Figure 5:**
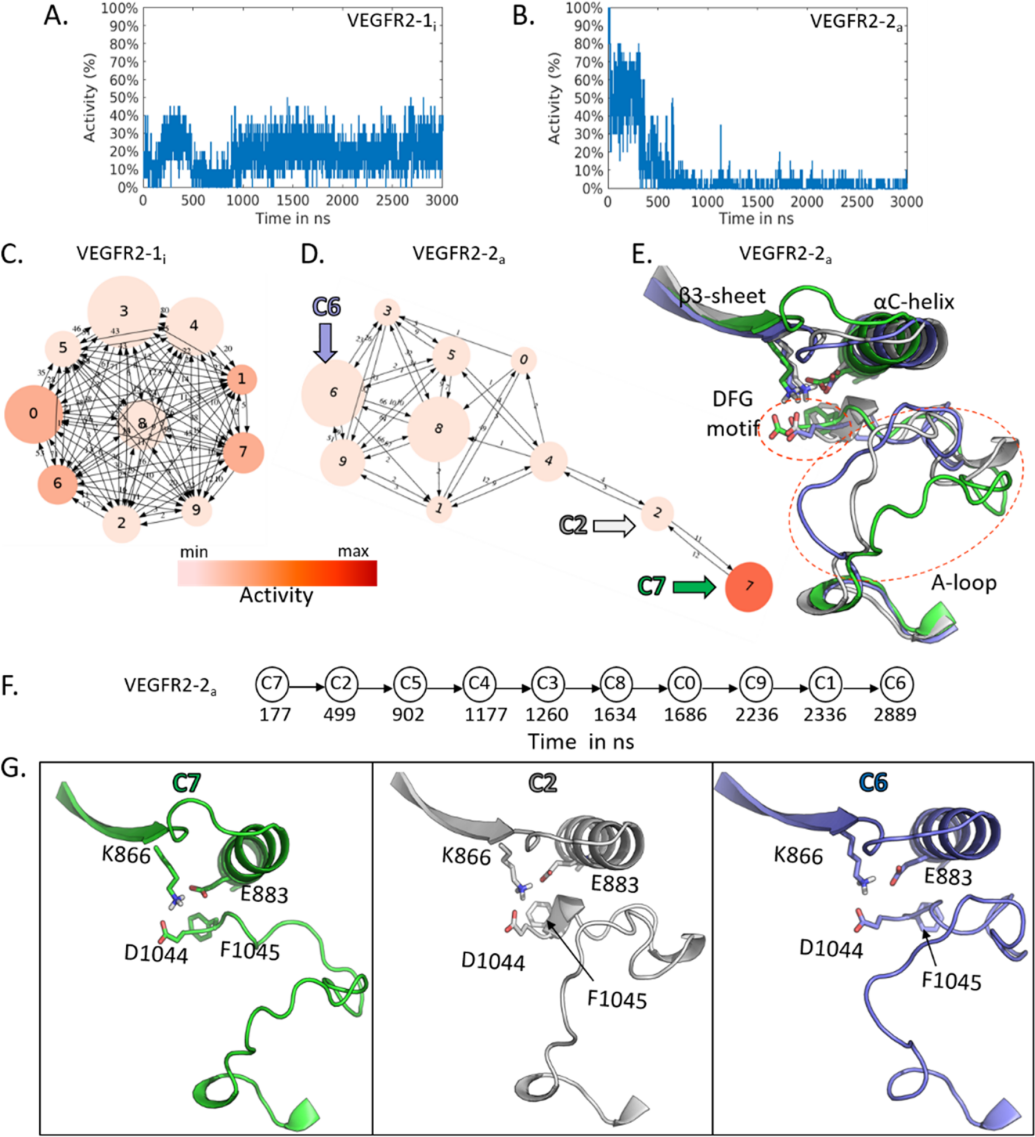
A-B. Activity probability estimation along the MD trajectories of VEGFR2 kinase, namely VEGFR2-1_i_ and VEGFR2-2_a_ (Table S1, no. 23 and no. 43, PDB ID 3vo3 and 3cjg, respectively). C–D. Cluster analysis of VEGFR2-1_i_ and VEGFR2-2_a_. Cluster medoids are colored according to the activity value. E. Superposition of representative conformations from C7 (green), C2 (white), and C6 (blue) of VEGFR2-2_a_. F. Time evolution over the clustering graph. G. Structural comparison of the A-loop and the “DFG-motif” among C7 (open), C2 (intermediate), and C6 (partially closed). Side chains of K866, E883, D1044, and F1045 are displayed in sticks (residue numbering according to PDB ID 3cjg).

We considered a pair of inactive and (putatively) active forms of VEGFR2 kinase domain, namely VEGFR2-1_i_ (PDB ID 3vo3)^49^ and VEGFR2-2_a_ (PDB ID 3cjg)^50^ VEGFR2-2_a_ was deemed active by the activity predictor, but this information was not available in the deposited structure (PDB ID 3cjg).^50^ We classified each MD-generated conformation for both trajectories (VEGFR2-1_i_ and VEGFR2-2_a_) with Kinconform^30^ (Figure 5B). VEGFR2-1_i_ oscillated between 0% and 40% of the activity probability at the beginning of the simulation and reached stability (∼20% activity) after 1 μs (Figure 5A). The starting structure’s initial activity value was 5%, and the average activity during the trajectory was 17.7% (Table S1, no. 23). Overall, the simulated conformations for this kinase mainly sampled inactive conformations. The VEGFR2-1_i_ trajectory was stable at a level of ∼0.2–0.25 nm RMSD compared to the starting structure and was thus considered to be structurally converged (Figure S3, no.23). The literature reports this kinase as “inactive”,^49^ in agreement with our findings: the “DFG-out” conformation was preserved, and the A-loop was in the inhibitory conformation. In contrast, the initial conformation of VEGFR2-2_a_ was classified as 100% active. This structure switched to the fully inactive form after 600 ns of simulation time (Figure 5B). Subsequently, it continued to sample inactive conformations until the end of the simulation at 3 μs (Figure 5B). For this kinase system, we reconstructed some residues (see Table S1) on the A-loop and 3 residues on the P-loop because they were missing from the X-ray crystal structure. Therefore, relatively long simulation times (on the order of 2 μs) were needed to fully equilibrate a reconstructed loop and determine the activity level in a stable way. Despite the fact that we observed this peculiar finding only for this kinase, this demonstrates that significant relaxation times might be required before any full production step in order to obtain robust results, particularly with free energy computations. Additionally, one can estimate the correct equilibration time using an activity score in order to objectively decide when to stop equilibration. This could be further extended to other systems (e.g., GPCRs) and other scores that capture some key information beyond the usual RMSD value.

The cluster analysis revealed only the inactive conformation of VEGFR2-1_i_ (Figure 5C), in agreement with activity plots calculated with the ML classifier. Indeed, all conformations can easily interconvert, even among the most populated clusters (i.e., C0, C3, C8). VEGFR2-2_a_ kinase explored mostly inactive conformations with an exceedingly small population of active states, as shown by the color-coded representation of the clusters (Figure 5D).

Out of ten clusters, only C7 presented an active conformation (55%) and remained isolated from the rest of the graph. To understand the structural differences, we superimposed the medoids of C2 and C6 on C7 (Figure 5E). C6 was highly linked to clusters C0, C1, C3, C5, C8, and C9, forming a clique. C4 behaved as a hub through which the C7 and C6 populations interconverted. The structural comparison revealed that the deviations were mainly associated with the DFG-motif and A-loop, as represented by the red circles (Figure 5E). The conformation associated with C7 represents an “open state” (Figure 5G, in green), whereas the corresponding conformer of C6 reveals a “partially closed state” of the kinase domain (Figure 5G, in blue). Notably, the “open state” facilitates the active conformation, whereas the “closed state” favors an inactive conformation. A small population of intermediate states was also found in C2 (Figure 5D). The conformation from C2 has an intermediate orientation of the phenyl ring of DFG F1045 as well as the A-loop (Figure 5G, in white), when compared to C6 and C7. These findings, together with the activity plot, show a coherent picture of partial inactivation during the MD run (see also Figure S5 for further structural comparisons of clusters).

### Insulin Receptor Kinase (IRK)

**Figure 6:**
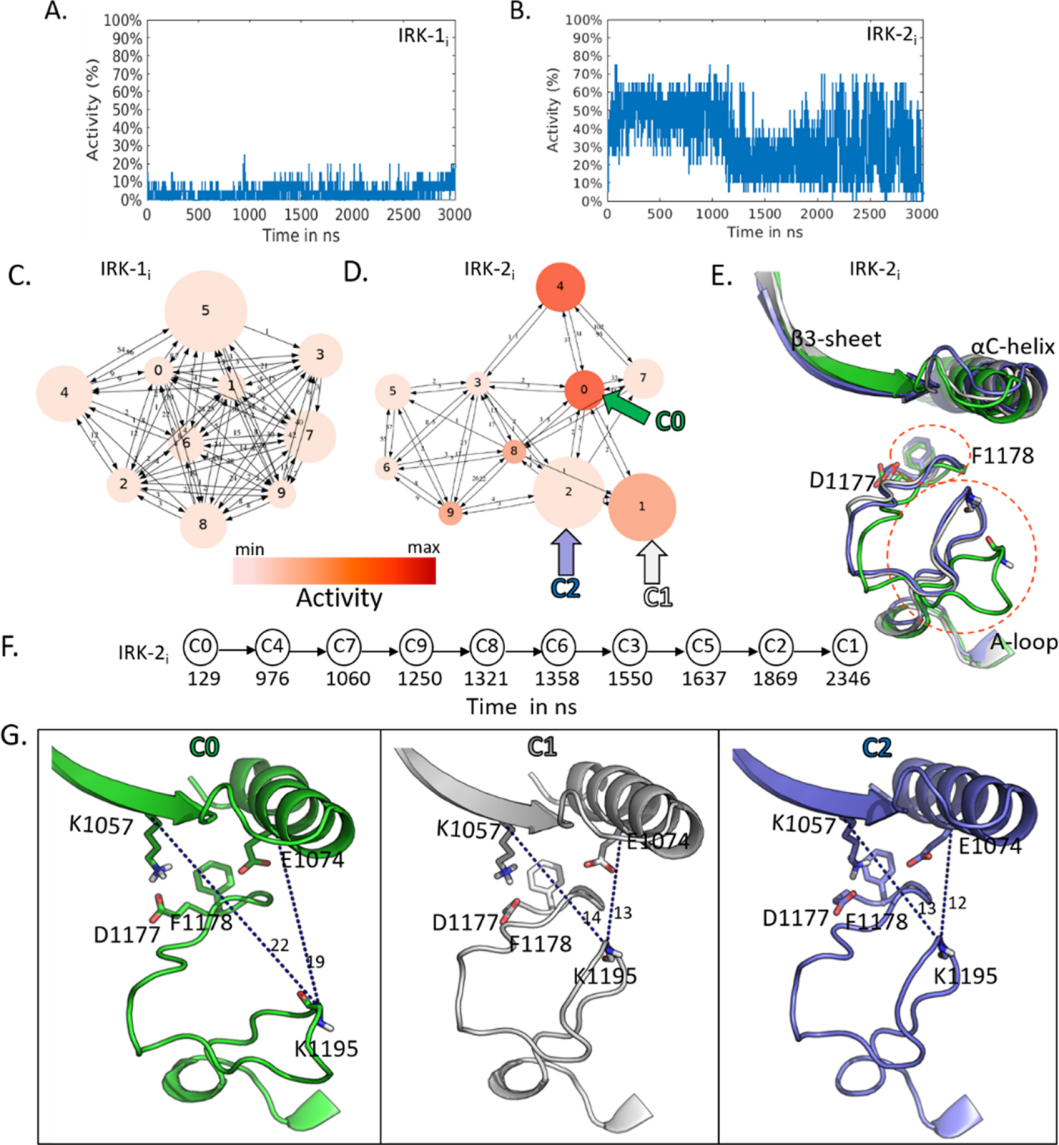
A-B. Activity probability estimation along the MD trajectories of IRK, IRK-1_i_, and IRK-2_i_ (Table S1, no. 10 and no. 41). C–D. Cluster analysis and representative conformations of IRK-1_i_ and IRK-2_i_. E. Superposition of the representative conformations from cluster C0, C1, and C2 of IRK-2_i,_ in green, white, and blue, respectively. F. Time evolution over the clustering graph. G. Structural comparison of the A-loop among C0 (“partial open”), C1 (“intermediate”), and C2 (“closed”) of IRK-2_i_. F1178, and D1177 belong to the “DFG-motif”. Dotted lines in blue indicate the distance of A-loop from K1057 (β3-strand) and E1074 (αC-helix) (residue numbering according to PDB ID 5hhw).

For the IRK (Figure 6A), we considered an apo (inactive) crystal structure in the unphosphorylated form (IRK-1_i_, PDB ID 1irk)^51^ and a second inactive structure in complex with an inhibitor (IRK-2_i_, PDB ID 5hhw,^34^ see Figure S6 for comparison). From the 3-μs-long simulation for IRK-1_i_, all conformations were predicted to be structurally inactive. The average activity was predicted to be 3.8% (Figure 6A, Table S1, no. 10), and the whole trajectory was stable in both the activity plot and RMSD (Figure S3, no.10). The IRK-2_i_ cocrystal was reported as inactive^34^ because of the presence of an inhibitor, which we removed for the simulation. However, IRK-2_i_ also sampled some partially active conformations (50%–65% activity, Figure 6B). The activity percentage of the initial conformation was estimated to be 10%, whereas the average activity along the whole trajectory was 33.9% (Table S1, no. 41). The MD simulation sampled conformations with partial activity (activity ranges between 40% and 65%) for the first ∼1 μs. After ∼1.3 μs, however, the sampled conformations were all inactive (Figure 6B), with an activity of 10–30%. Interestingly, the system again started sampling partial active conformations (50% −65% activity) after 2 μs until the simulation end.

For IRK-1_i_, the clustering graph was fully connected, demonstrating that the conformations could easily interconvert (Figure 6C). Each medoid had a predicted activity level ranging between 0% and 10% only. For IRK-2_i_, the cluster analysis mainly revealed two well-populated clusters, C1 and C2, located close together in the RMSD space (Figure 6D).

Both clusters sampled structurally inactive states of the kinase. C0 and C4 were identified as moderately active, with C4 having higher activity values (around 55%). We compared the representative conformations of C0, C1, and C2 to clarify the activity switching in correlation with the structural differences (Figure 6E). The cluster centers are the structures at simulation times 129, 2346, and 1869 ns, respectively, and bear the main differences in the A loop (Figure S6). This structural evidence indicates that A-loop switching is mainly associated with the activity status. The C0 cluster, showing 50% activity (Table S2, no. 41), represents a partially closed configuration of the ATP binding pocket, in which the DFG aspartate is pointing out toward the cleft. At the same time, the phenylalanine resides inside the pocket (Figure 6G, in green, Figure S7). Moreover, in the corresponding medoid, the K-E salt bridge is lost. On the other hand, the C2 medoid (Figure 6G) is an inactive conformation of IRK-2_i_ (5% of estimated activity only, Table S2, no. 41). Finally, the C1 medoid (Figure 6G, in white) shows an intermediate conformation of the A-loop compared with the C0 and C2 clusters (Figure 6G, in green and blue, respectively). Indeed, the medoid structure extracted from C1 showed 40% activity.

### Bruton’s Tyrosine Kinase (BTK)

**Figure 7:**
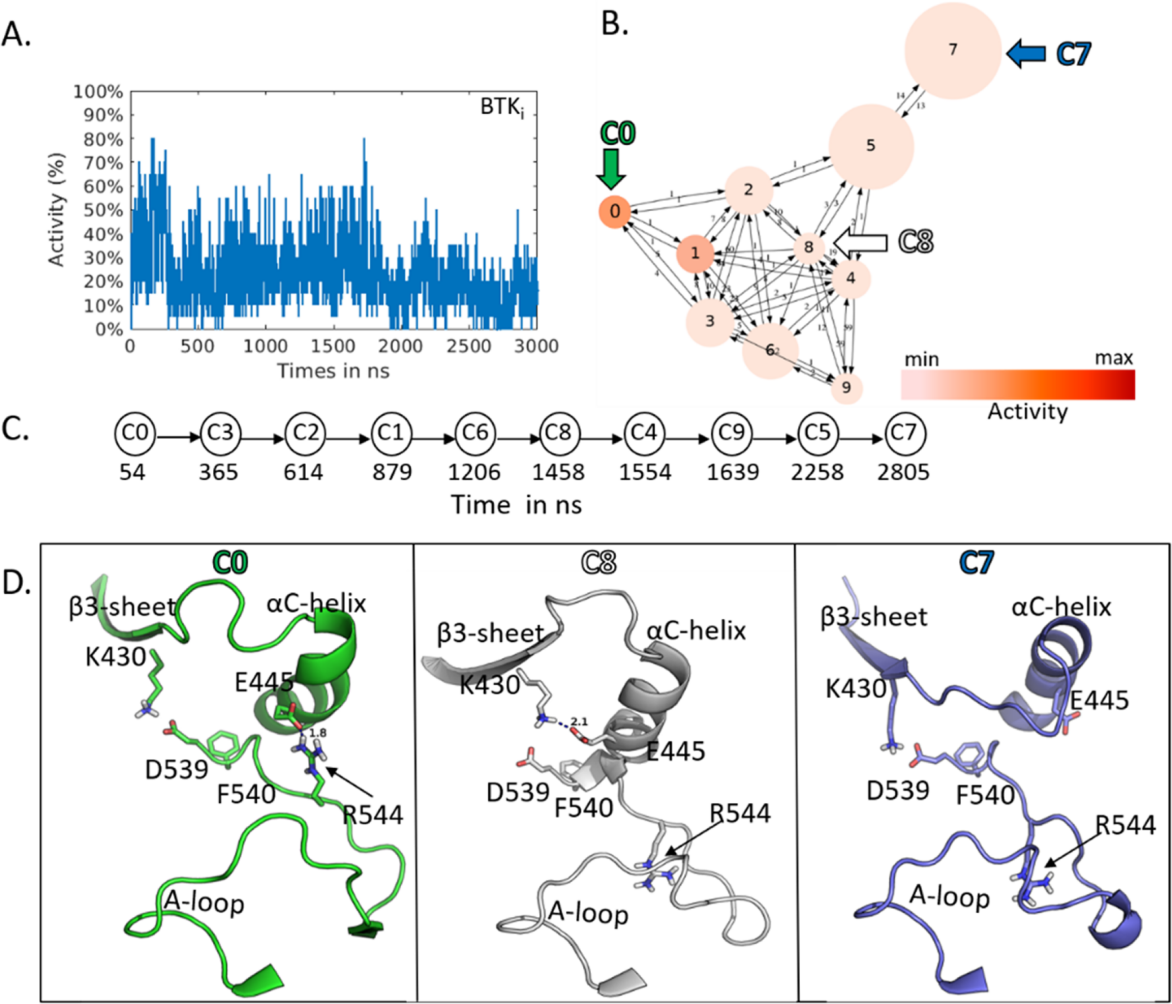
A. Activity analysis of BTK_i_considering the MD trajectory. B. Cluster analysis of BTK_i_. Each medoid is labeled according to the activity. C. Schematic representation of the time evolution of clusters. D. Representative conformations from C0, C7, and C8 show the structural changes of BTK_i_ over time. Secondary structures in green, white, and blue represent the corresponding conformations of C0, C8, and C7 (decreasing activity order). Orientation of the side chains of K430, E445, D539, F540, and R544 are represented in sticks (residue numbering according to PDB ID 1k2p). A. Activity analysis of BTK_i_ considering the MD trajectory. B. Cluster analysis of BTK_i_. Each medoid is labeled according to the activity. C. Schematic representation of the time evolution of clusters. D. Representative conformations from C0, C7, and C8 show the structural changes of BTK_i_ over time. Secondary structures in green, white, and blue represent the corresponding conformations of C0, C8, and C7 (with decreasing activity order). Orientation of the side chains of K430, E445, D539, F540, and R544 are represented in sticks (residue numbering according to PDB ID 1k2p).

Only a putatively inactive conformation of BTK was available (BTK_i_). The simulation mostly sampled inactive conformations, along with a few partially active states (Figure 7A). The average activity of this kinase was estimated at 22.8% (Table S1, no. 42), while the initial activity was estimated at 35%. A few conformations were obtained with 50–60% activity in the first 2 μs of MD simulation. In the final 1 μs, the system mainly sampled inactive states (Figure 7A). The PDB structure (1k2p) and the literature reported BTK_i_ as an unphosphorylated and inactive kinase, revealing a unique mechanism of activation.^52^ According to the literature, the A-loop had an active-like noninhibitory conformation, whereas the αC-helix adopted an inactive conformation (Figure S8). Thus, BTK could be a special case relative to the other inactive TKs, where the A-loop appears in a closed state. Moreover, the crystal structure does not show the possibility of a salt bridge formation between the K430 and E445 residues,^52^ which is considered one of the crucial hallmarks for BTK activation.^53^ Rather, E445 seems to stabilize the R544 side chain of the A-loop to preserve its open conformation, preventing formation of the K430-E445 salt bridge^52^ (Figure S9).

Of the 10 clusters, only C0 was captured as a small population of partially active conformations (Figure 7B). The C0 cluster is quite small (Figure 7B) compared to the others, which is in line with the activity plot. From the time mapping, C0 and C7 represent the conformations of BTK_i_ at the beginning (54 ns) and at the end (2805 ns) of the MD simulation, respectively (Figure 7C). The C0 medoid shows 40% activity and the E445-R544 salt bridge (Figure 7D, in green). On the other hand, C8 restores the inactive population of BTK_i_ (1458 ns simulation time). In the middle of the simulation, the side chain of R544 flips toward the TK catalytic site, and E445 (αC-helix) is free to interact with K430 (β3-sheet; Figure 7D, in white). The cluster analysis also captured a large medoid, C7, where E445 loses contact with K430, and R544 is flipped as in C8 (Figure 7D, in blue). The superposition of C0, C8, and C7 revealed another significant change in the conformations of BTK_i_ where the side chain conformation of D539 of the DFG-motif was observed more inside the ATP-site for C0 than C7 (Figure S9).

## Conclusions

In this contribution, we report on a family wide analysis of tyrosine kinases with a focus on some of their particular activity patterns. We ran a massive MD campaign to understand the flexibility–activity relationships in a large set of representative tyrosine kinases. Physics-based simulation, high-performance computing, and ML were the key tools. Analysis of the pockets highlighted the increased flexibility of the inactive structures. This finding confirms previous observations with computations performed here for the first time on a large, dynamic, and complete scale. We show that flexibility (even of a single residue) may help predict activity, with a validation-set accuracy higher than random chance. Key amino acids are the arginine or lysine residues found in all 43 simulated TKs, and located at the DFG+3 position on which fluctuations we were able to detect a semiquantitative threshold. Considering the multiple-sequence aligned 497 human kinases,^48^ this DFG+3 residue is either Lys or Arg in >90% of the TK family, in 66% (41/62) of the AGC family, in 68% (44/65) of the CMGC family, in 100% (11/11) of the NEK family, in 58% (7/12) of the CK1 family, in 40% of the TKL (17/42) and in 73% of the CAMK (37/51) families. An ablation analysis showed that the fluctuations from DFG+1 to DFG+3 are generally related to activity, which clearly indicates the activation segment as the key player. This finding was obtained in a fully unbiased way apart from the required loops’ reconstructions. The present rule is minimalistic and cannot fully account for activity, yet it avoids overfitting and gives an indication and a recognizable fingerprint. From a drug discovery standpoint, this analysis identifies an opportunity to target inactive forms when designing new TK ligands. Indeed, for the inactive conformations, we found more opportunities in terms of the presence, volumes, and potential allostery of pockets. It is unclear if targeting the active or inactive form is the proper choice for a given disease. However, we know that targeting inactive forms often leads to increased selectivity.^54^ Notably, by analyzing X-ray crystal structures of active and inactive kinases, a related “selectivity exploring flexibility” paradigm was proposed more than 20 years ago.^54^ Here, however, differently from that analysis, we have shown that inactive forms tend to be different from each other, and they also bear an intrinsically higher fluctuation propensity. Moreover, we fully characterized the flexibility in a pocket specific way which is unprecedented to Authors’ knowledge.

Lastly but possibly more relevantly, the collected trajectories could be used as an atlas of conformations and pockets for virtual screening and docking campaigns. The data set of collected medoids is publicly available via the IIT Dataverse (see Data Availability Statement and Software Availability).

These findings are valid for TKs. However, in accordance with recent computational findings,^39^ we can conjecture that the same flexibility pattern may hold for the kinase family in general and that DFG+3 is a key residue for determining activity. Lastly, our large-scale approach is entirely hypothesis-free and heavily data-driven, so it could be translated to other kinase families or even to other proteins of biological and pharmaceutical interest (e.g., G-protein coupled or nuclear receptors).

## Methods

### Simulation Setup

To set up the simulations, we used the BiKi Life Sciences software suite^55^ and the Amber 14 force field.^56^ We parametrized the post-translationally modified phosphotyrosine^57^ via the database at http://amber.manchester.ac.uk. Missing loops were rebuilt using the BiKi Life Sciences loop rebuilding tool or Schrödinger Maestro (Release 2020–3: Maestro, Schrödinger, LLC, New York, NY, 2020). All simulations were run via Gromacs 4.6.1.^58,59^ Electrostatics was managed using the Particle Mesh Ewald^60,61^ for long-range interactions and with a cutoff of 1.2 nm. Minimization was done via the steepest descent method, and equilibration followed the standard BiKi protocol, which encompasses 3 NVT steps of 100 ps each and a final NPT step of 1 ns.

To analyze the systems, we used three different techniques: (i) an existing ML classifier to estimate the activity/inactivity of each molecular dynamics configuration^30^ (the activity plots for all analyzed TKs are provided in Figure S2); (ii) a clustering of structures via the k-medoids algorithm^62^ (the clustering networks of all the TK are provided in Figure S10); and (iii) the Pocketron^33^ algorithm to study the pockets and their cross-talk.

### Pocket Analysis

We ran this analysis with Pocketron.^33^ This tool is available in the BiKi Life Sciences suite (www.bikitech.com),^55^ and can track pockets’ dynamical behaviors along an MD trajectory. For each pocket, the Pocketron algorithm can estimate the communication pattern among the pockets and provide a corresponding “Pockets Network” map of the system. The detection of the pockets at the frame level is done by NanoShaper 0.7 (available at https://gitlab.iit.it/SDecherchi/nanoshaper).^63^ To compute the pocket network maps, Pocketron was applied to all MD trajectories of the 43 kinases with an interval of 100 ps between consecutive frames. Initially, all the solvent molecules were removed to analyze only the protein component, and the two default probe radii of 1.4 and 3 Å were used. This analysis also delivers “merging” and “splitting” events of each pocket during the simulation. Using the merge and split matrices,^33^ after making them symmetric through averaging, we prepared a connection matrix γ by averaging the corresponding values of the merge and split matrices α and β:
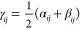


We computed two of these γ matrices: one for the active set and one for the inactive set. Lastly, we computed the connections’ distributions on the entries of this matrix by considering each row at a time (as each row corresponds to a single pocket).

To evaluate if the means of the volume distributions and the number of connections for the different pockets were statistically different for the active and inactive kinases, we used Welch’s *t* test^38^ or unequal variances *t* test. This is a generalization of Student’s hypothesis test statistic^64^ for samples with unequal variances and/or unequal sample sizes, as in the cases considered in our analysis.

### Activity Prediction Based on Fluctuations

To perform this analysis, we initially created a data set (“X” matrix) based on RMSF values estimated from the trajectories by removing the first 500 ns as equilibration time. To prepare the matrix, a sequence alignment step was needed. We first retrieved the UniProt sequences (www.uniprot.org) of each of the 43 TKs of interest, then performed a multiple sequence alignment using Clustal Omega^65^ (Figure S1). Finally, a 43 × 223 matrix of RMSF values (based on the backbone atoms only) was generated corresponding to each of the 223 residues for all 43 TKs (residue indices of all proteins are in Supporting Data File 1). The RMSF values of all the corresponding aligned residues (Figure S1) were computed via Gromacs 2019.4.^58^ To determine an activity label, “y”, for each kinase, we estimated the average activity value predicted by Kinconform 1.0^30^ along the MD trajectories. This tool can distinguish kinase conformations as active/inactive based on the orientation of the activation segment alone.^30^ This prediction was coherent with the inactivity/activity label reported in the literature, as such the “y” vector was essentially coming from experiments. Overall, the simulations derived X matrix was used to predict the experimental values in y as often happens in MD (e.g., free energy estimations).

To project the matrix, we used the t-SNE method,^44^ and a classification tree for building the classifier.^44^ To make it interpretable, we constrained the solution to contain only one rule only. We employed Python 3.7 and the Scikit-learn library (version 0.22.1 on win64)^66^ to support this activity using all default values (random_state = 0 for t-SNE). To estimate the error, we split the data into training and validation sets with various percentages, obtaining balanced accuracies between 70% and 80%. The balanced accuracy is the mean of the errors in the positive and negative classes, hence giving a more reliable measure than classical accuracy, which is heavily influenced by class imbalance. The balanced accuracy is, therefore, the mean of the sensitivity and specificity. Then we fixed at 0.7 the ratio between training and validation set and repeated the random split 100 times, obtaining a final balanced accuracy 72.96% ± 11.78%.

### Cluster Analysis

The MD trajectories were clustered through the k-medoids algorithm^62^ implemented in the BiKi Life Sciences suite.^55,67^ For cluster generation, we used the RMSD matrix of the entire segment of the A-loop, including the DFG-motif. The set of residues considered to perform the cluster analysis is highlighted in the inset of Figure 1. Table S2 reports the activity percentage of each kinase conformation corresponding to each cluster. The side chain of D1177, F1178, and G1179 (PDB ID 5hhw)^34^ were selected along with the backbone of the rest of the A-loop segment to be coherent with the Kinconform features.^30,68^ Indeed, the authors included the χ angle of F1178 and G1179 side chains (according to PDB ID 5hhw)^34^ followed by several φ, ψ, and pseudodihedral angles through the Cα atoms of the A-loop.^69^ We ran the clustering, always setting the number of clusters to 10 (Figure S10). The size of each cluster circle encodes its cardinality, and the number on the edges encodes interconversions between the clusters. Each medoid is the most central frame of the cluster.

## Data Availability

The medoids data which supports the findings are available at https://doi.org/10.48557/UARU6J. The resulting files from the pocket analysis on the 43 TKs MD trajectories via Pocketron are available at https://doi.org/10.48557/Z5E3YG. The full trajectories are available upon request.

## Supporting Information Available

Supplementary text: System preparation and MD simulations; RMSD analysis. Tables S1 to S4: List of kinases, predicted activity probability, number of connections and number of pockets identified. Figures S1 to S10: RMSF, estimated activity probability (using Kinconform), RMSD, DFG+3 interaction graphs, several snapshots for structural comparisons, and clustering analysis. (PDF)
Supporting Data File 1. Residue indices of the 43 TKs (CSV)

## Notes

The software used to rebuild the loops are the commercial software Schrödinger Maestro (Release 2020–3) and BiKi Life Sciences 1.5 (development version). All the simulations were prepared via BiKi Life Sciences 1.3.5 and run via Gromacs 4.6.1. The clustering and Pocketron analysis was performed via BiKi Life Sciences 1.3.5. Kinconform version used was 1.0 for the activity prediction. To support the fluctuation analysis we used, the Clustal Omega Web server for sequence alignment, Gromacs 2019.4 to build the RMSF matrix and the software available at https://gitlab.iit.it/SDecherchi/kinase_atlas for the machine learning analysis.

## ABBREVIATIONS

TKs: tyrosine kinases
MD: molecular dynamics
ML: machine learning
A-loop: activation loop
IRK: insulin receptor kinase
VEGFR2: vascular endothelial growth factor receptor 2
BTK: Bruton’s tyrosine kinase
RMSD: root-mean-square deviation
RMSF: root-mean-square fluctuation.
