# Supplementary material for: Molecular Dynamics and Machine Learning Give Insights on the Flexibility–Activity Relationships in Tyrosine Kinome
Source: J Chem Inf Model. 2023 Jul 18;63(15):4814–26. doi: 10.1021/acs.jcim.3c00738 (PMC10428216; doi:10.1021/acs.jcim.3c00738)
Supplement: Supplementary file 1 — ci3c00738_si_001.pdf [file ci3c00738_si_001.pdf]

Supporting Information

Molecular dynamics and machine learning give insights on the flexibility-activity relationships in tyrosine kinome

Sarmistha Majumdar[1°], Francesco Di Palma[1°], Francesca Spyrakis[2*], Sergio Decherchi[3*], Andrea Cavalli[1,4]

1 Computational & Chemical Biology, Fondazione Istituto Italiano di Tecnologia, Via Morego 30, I-16163 Genova, Italy
2 Department of Drug Science and Technology, University of Turin, via Giuria 9, 10125, Turin, Italy
3 Data Science and Computation, Fondazione Istituto Italiano di Tecnologia, Via Morego 30, I-16163 Genova, Italy
4 Department of Pharmacy and Biotechnology, University of Bologna, Bologna, Italy.

° co-first Authors
* co-corresponding Authors

This PDF file includes:

Supplementary Text

1. Systems preparation and Molecular Dynamics (MD) simulations
2. RMSD analysis

Tables S1 to S4

Figures S1 to S10

Supplementary Text

1. System preparation and Molecular Dynamics (MD) simulations

All the simulated systems considered in this work belong to the tyrosine (TYR) kinase family proteins. We selected, limited by available computational power, several X-ray crystal structures with resolution at least of 3Å, available in the Research Collaboratory for Structural Bioinformatics (RCSB) database (www.rcsb.org). In case of co-complexes we removed all the ligands and considered only the protein part. Missing loops of some of the kinases (as detailed in Supporting Table S1) were reconstructed using the Maestro interface of Schrödinger (Release 2020-3: Maestro, Schrödinger, LLC, New York, NY, 2020) or the BiKi Life Sciences software suite. After reconstruction of the missing parts loops, the BiKi Life Sciences software suite[1] was used to immerse each systems into a water box using the TIP3P[2] water model. Neutrality of the systems was ensured by replacing the water molecules with an appropriate number of Cl^- and Na^+ ions. All the systems were parameterized with the Amber14SB forcefield[3] and were simulated via Molecular Dynamics using GROMACS version 4.6.1[4,5]. They were equilibrated with a target temperature of 300 K and a target pressure of 1 bar. The velocity rescaling thermostat[6] was employed to heat the systems gradually from 100 K up to 300 K in 3 steps (200 ps each). The last equilibration step was done in the NPT ensemble for 1000 ps using the Parrinello-Rahman barostat[7]. After equilibration, the production runs were performed in the NVT ensemble. The equations of motion were integrated with a time step of 2 fs and hydrogen bonds were constrained using the LINCS algorithm[8]. The particle mesh Ewald (PME) method[9,10] (hence using periodic boundary conditions) for long-ranged electrostatic interactions was set with a cutoff of 11 Å.

2. RMSD analysis

To check the equilibration time of each system we calculated the average root mean square deviation (RMSD) of all the backbone atoms with respect to the first frame. On average RMSD values did not drift much compared to the initial frame, except few cases. High deviations were observed only when associated to the motion of the unstructured regions. The analysis was done via the GROMACS tools set.

Table S1. List of kinases considered in our investigation. We report: kinase abbreviation and reference PDB structure in parenthesis, total sampling time, predicted initial and average activity along the simulation, reference paper to judge initial activity and if we rebuilt missing loops (and in parenthesis the number of the residues modeled in the activation loop).

Index	Kinase (PDB ID)	Time (μs)	Initial Activity (%)	Average Activity (%)	Reference Paper	Missing Loop Reconstruction
1	CSK (1byg)	3	0	12.5	Inactive[11]	Yes (12)
2	EGFR-1 (2jit)	3	5	4.3	Inactive[12]	No
3	EPHA2-1 (5ek7)	3	0	17.6	-	No
4	EPHA3-1 (4twn)	2.79	15	3.8	Inactive[13]	Yes (15)
5	EPHB4 (2vww)	3	30	25.6	-	Yes (24)
6	FGFR1-1 (3c4f)	2.88	25	9.3	Inactive[14]	No
7	HCK (5h0b)	2.58	0	2.5	-	No
8	HER3 (4otw)	3	0	0.8	-	No
9	IGFR1-1 (1p4o)	3	15	9.8	Inactive[15]	No
10	IRK-1_i (1irk)	2.9	5	3.8	Inactive[16]	No
11	ITK (4hcu)	3	0	1.2	Inactive[17]	No
12	JAK2-1 (3ugc)	3	10	11.2	-	Yes (12)
13	KIT-1 (3g0e)	3	15	21.8	Inactive[18]	No
14	MET-1 (4r1v)	3	0	5.7	-	No
15	MET-2 (5hti)	2.07	0	15.8	-	Yes (5)
16	PYK2 (3cc6)	3	0	1.8	-	Yes (9)
17	RON (3pls)	3	15	13.6	-	No
18	SRC-1 (2src)	1.58	10	1.6	Inactive[19]	No
19	SYK-1 (3tub)	2.48	5	4.2	Inactive[20]	Yes (19)
20	TIE2-1 (1fvr)	3	10	5.7	Inactive[21]	Yes (4)
21	TIE2-2 (3l8p)	3	10	20.0	-	No
22	TRKA (4pmm)	2.92	0	1.6	Inactive[22]	No
23	VEGFR2-1_i (3vo3)	3	5	17.7	Inactive[23]	No
24	EGFR-2 (1m17)	2.9	80	92.5	Active[24]	No
25	EPHA2-2 (1mqb)	3	100	94.9	Active[25]	Yes (17)
26	EPHA3-2 (4two)	1.48	100	92.0	Active[13]	Yes (15)
27	ERBB4 (3bce)	1.3	85	92.7	Active[26]	No
28	FES (3bkb)	3	100	95.2	Active[27]	No
29	FGFR1-2 (3gqi)	3	100	96.2	Active[28]	No
30	FGFR2 (1gjo)	3	100	92.3	-	No
31	IGFR1-2 (1k3a)	3	80	84.8	Active[29]	No

32	JAK1 (3eyh)	3	90	91.3	Active[30]	No
33	JAK2-2 (6bbv)	1.8	85	93.7	-	No
34	JAK3 (5lwm)	3	95	96.8	-	No
35	KIT-2 (1pkg)	3	95	96.4	Active[18]	No
36	LCK (3lck)	3	100	96.1	Active[31]	No
37	LYN (3a4o)	3	90	85.5	-	Yes (9)
38	SRC-2 (1y57)	2.68	95	88.5	Active[32]	No
39	SYK-2 (5tt7)	3	100	95.4	Active[33]	No
40	TYK2 (3lxn)	3	95	94.0	Active[34]	Yes (3)
41	IRK-2$_i$ (5hhw)	3	10	33.9	Inactive[35]	No
42	BTK$_i$ (1k2p)	3	35	22.8	Inactive[36]	No
43	VEGFR2-2$_a$ (3cjg)	3	100	8.1	-	Yes (14)

Table S2. List of predicted activity probability (%) of the conformations obtained from cluster analysis. The ten obtained clusters are indexed from 0.

Index	Kinase	C0 (%)	C1 (%)	C2 (%)	C3 (%)	C4 (%)	C5 (%)	C6 (%)	C7 (%)	C8 (%)	C9 (%)
1	CSK	15	15	15	15	10	10	10	20	20	10
2	EGFR-1	0	5	5	0	5	0	5	5	5	5
3	EPHA2-1	25	15	20	15	30	30	30	25	20	5
4	EPHA3-1	0	5	0	5	5	5	5	0	0	5
5	EPHB4	25	25	30	30	20	35	25	20	15	20
6	FGFR1-1	0	10	10	10	0	0	0	5	0	0
7	HCK	0	5	0	5	0	10	5	0	0	0
8	HER3	0	0	10	0	5	0	0	0	0	0
9	IGFR1-1	5	10	15	15	5	15	15	10	20	10
10	IRK-1$_i$	10	5	10	0	0	5	0	10	0	0
11	ITK	5	5	0	5	0	5	5	0	0	0
12	JAK2-1	5	15	0	15	0	5	25	5	10	20
13	KIT-1	15	20	25	15	25	40	25	20	30	30
14	MET-1	5	0	10	0	0	10	0	5	5	15
15	MET-2	0	40	30	30	20	10	0	20	5	15
16	PYK2	0	0	0	0	10	0	0	0	0	0
17	RON	25	15	100	15	20	15	100	100	20	10
18	SRC-1	0	0	0	0	5	0	0	0	0	0
19	SYK-1	10	5	0	5	5	0	0	0	15	5
20	TIE2-1	10	0	5	0	0	0	10	5	5	5
21	TIE2-2	25	10	5	15	30	20	30	20	20	25
22	TRKA	0	0	0	0	5	5	0	0	0	0
23	VEGFR2-1$_i$	30	30	0	20	15	20	30	25	0	15
24	EGFR-2	80	100	95	85	95	100	100	95	100	100
25	EPHA2-2	100	100	90	95	85	80	95	100	100	100
26	EPHA3-2	75	100	95	100	100	100	80	85	90	95
27	ERBB4	95	95	95	95	100	95	95	100	95	95

28	FES	100	100	100	100	95	95	95	90	100	100
29	FGFR1-2	100	100	100	95	95	100	100	100	100	100
30	FGFR2	90	90	90	100	100	95	100	100	100	95
31	IGFR1-2	95	80	100	100	90	75	80	65	65	95
32	JAK1	90	100	100	85	70	100	100	95	85	100
33	JAK2-2	90	100	95	90	100	90	100	95	100	100
34	JAK3	95	100	100	100	100	100	100	95	95	100
35	KIT-2	95	100	100	100	95	95	95	90	95	90
36	LCK	95	100	100	100	100	90	100	95	100	100
37	LYN	90	80	85	85	80	90	75	85	95	90
38	SRC-2	85	95	90	95	95	80	80	95	90	85
39	SYK-2	100	100	100	90	95	95	100	100	100	100
40	TYK2	100	75	95	95	100	100	95	85	90	95
41	IRK-2$_i$	50	40	5	20	55	20	15	5	35	25
42	BTK$_i$	40	30	10	15	15	20	20	10	20	20
43	VEGRF2-2$_a$	0	0	0	0	0	0	0	55	0	0

Table S3. Number of connections (and corresponding probability) formed by ATP binding pocket obtained through Pocketron analysis[37].

**Index**	**Kinase**	**N. of connections**	**Probability (%)**	**Index**	**Kinase**	**N. of connections**	**Probability (%)**
1	CSK	28	63	23	VEGFR2-1$_i$	23	49
2	EGFR-1	25	58	24	EGFR-2	26	58
3	EPHA2-1	18	41	25	EPHA2-2	22	46
4	EPHA3-1	31	64	26	EPHA3-2	26	59
5	EPHB4	23	50	27	ERBB4	30	65
6	FGFR1-1	24	50	28	FES	19	50
7	HCK	17	46	29	FGFR1-2	20	41
8	HER3	21	60	30	FGFR2	25	55
9	IGFR1-1	25	47	31	IGFR1-2	21	40
10	IRK-1$_i$	26	49	32	JAK1	18	42
11	ITK	16	41	33	JAK2-2	19	41
12	JAK2-1	26	54	34	JAK3	24	54
13	KIT-1	21	51	35	KIT-2	20	46
14	MET-1	19	46	36	LCK	20	45
15	MET-2	31	63	37	LYN	23	56
16	PYK2	27	55	38	SRC-2	19	50
17	RON	22	50	39	SYK-2	23	47
18	SRC-1	13	40	40	TYK2	26	53
19	SYK-1	24	63	41	IRK-2$_i$	24	46
20	TIE2-1	27	54	42	BTK$_i$	20	49
21	TIE2-2	25	54	43	VEGFR2-2$_a$	25	55
22	TRKA	25	54				

Table S4. Number of total pockets identified in each kinase domain with time persistency higher than 30%.

Index	Kinase	N. of pockets	Index	Kinase	N. of pockets
1	CSK	7	23	VEGFR2-1_i	7
2	EGFR-1	7	24	EGFR-2	5
3	EPHA2-1	9	25	EPHA2-2	10
4	EPHA3-1	11	26	EPHA3-2	11
5	EPHB4	8	27	ERBB4	9
6	FGFR1-1	9	28	FES	5
7	HCK	5	29	FGFR1-2	10
8	HER3	6	30	FGFR2	10
9	IGFR1-1	9	31	IGFR1-2	11
10	IRK-1_i	10	32	JAK1	8
11	ITK	8	33	JAK2-2	7
12	JAK2-1	6	34	JAK3	12
13	KIT-1	8	35	KIT-2	7
14	MET-1	6	36	LCK	7
15	MET-2	9	37	LYN	4
16	PYK2	5	38	SRC-2	10
17	RON	8	39	SYK-2	8
18	SRC-1	8	40	TYK2	8
19	SYK-1	5	41	IRK-2_i	7
20	TIE2-1	12	42	BTK_i	8
21	TIE2-2	6	43	VEGFR2-2_a	7
22	TRKA	9			

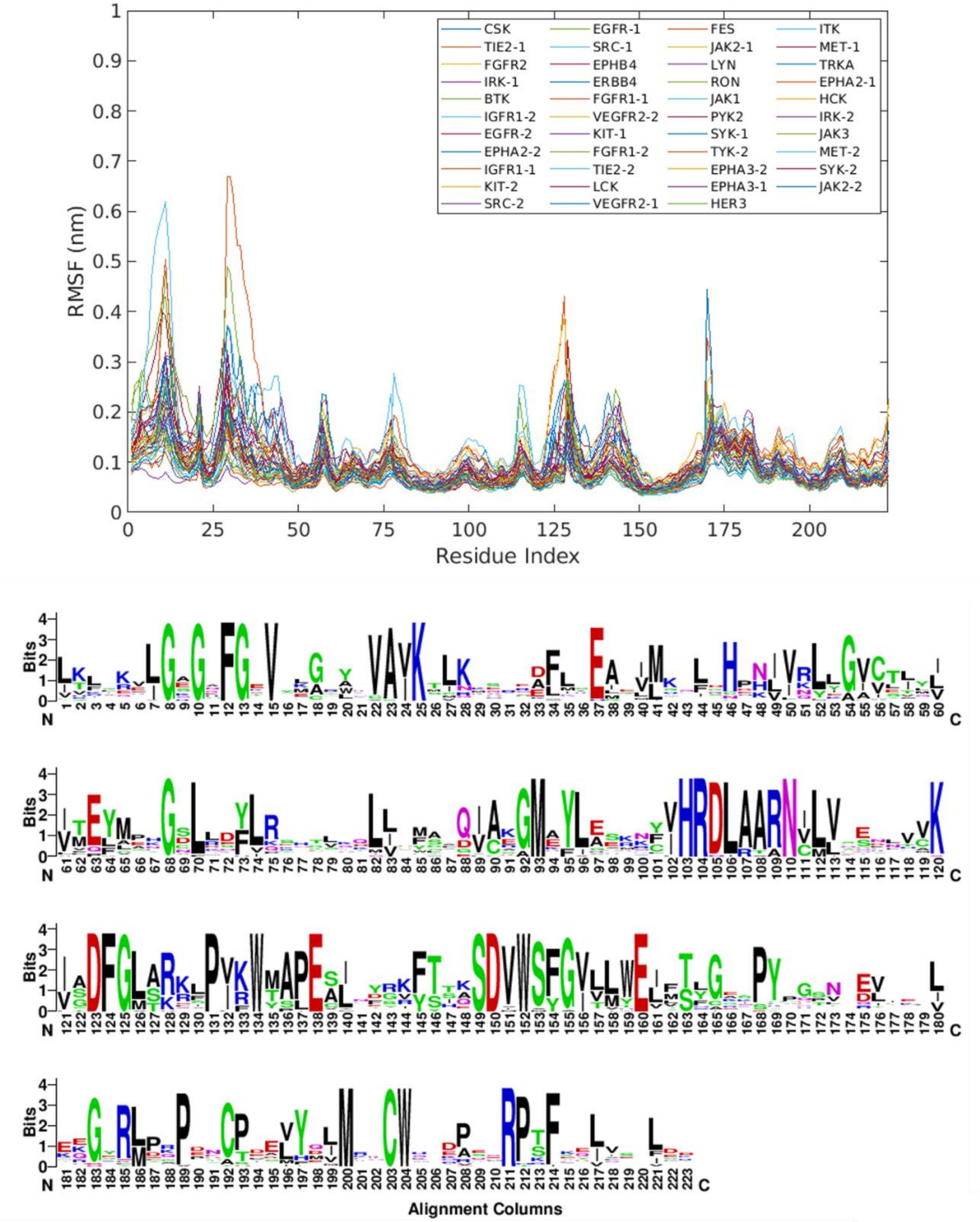

Figure S1. Root mean square fluctuations (RMSF) of kinase residues (top) using the whole trajectories. We report the mapping of indices in the Supporting Data File 1. A sequence logo has been created by using WebLogo[38] (below).

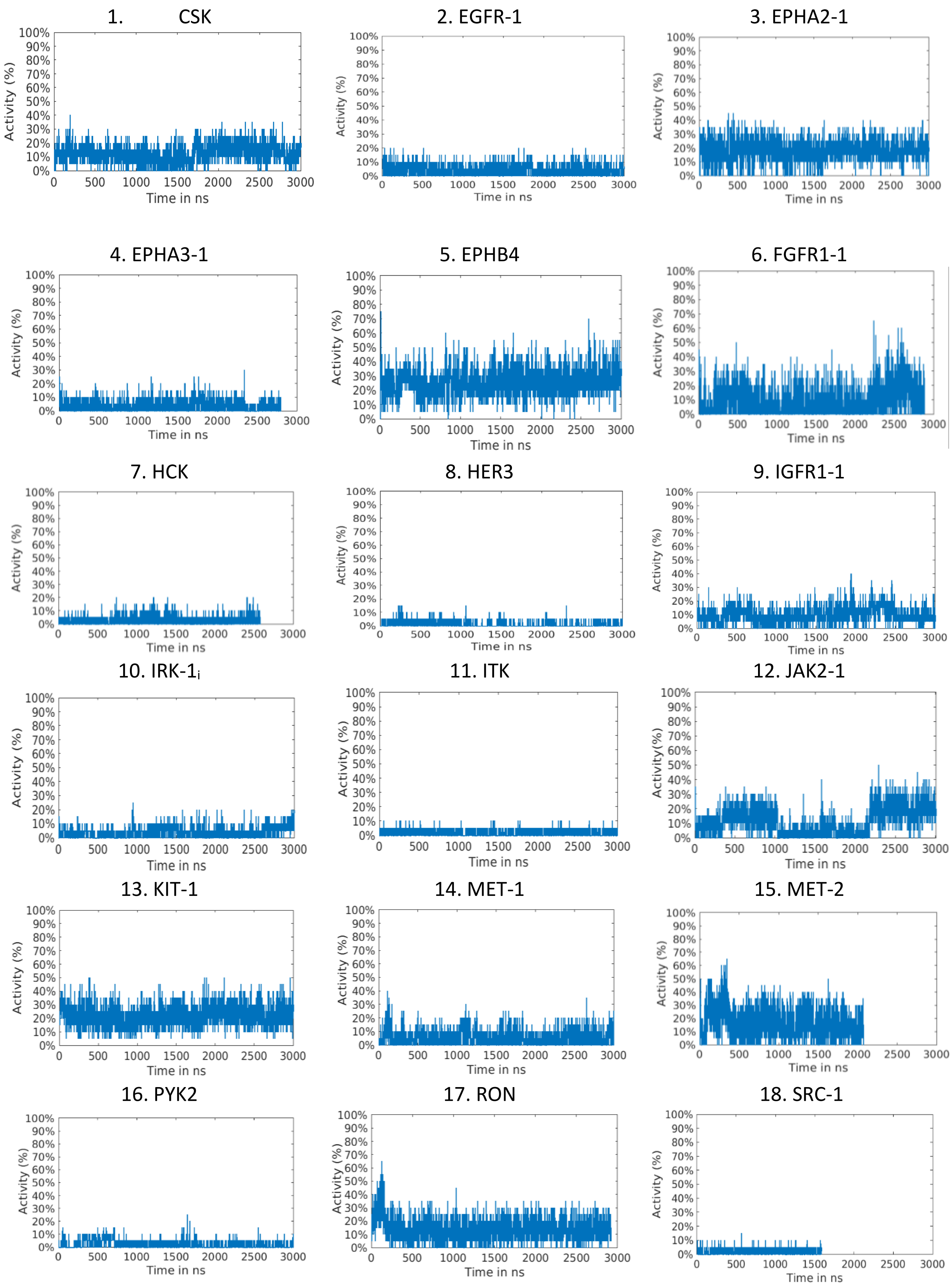
1. CSK
2. EGFR-1
3. EPHA2-1
4. EPHA3-1
5. EPHB4
6. FGFR1-1
7. HCK
8. HER3
9. IGFR1-1
10. IRK-1i
11. ITK
12. JAK2-1
13. KIT-1
14. MET-1
15. MET-2
16. PYK2
17. RON
18. SRC-1
Activity (%)
Time in ns
0% 10% 20% 30% 40% 50% 60% 70% 80% 90% 100%
0 500 1000 1500 2000 2500 3000

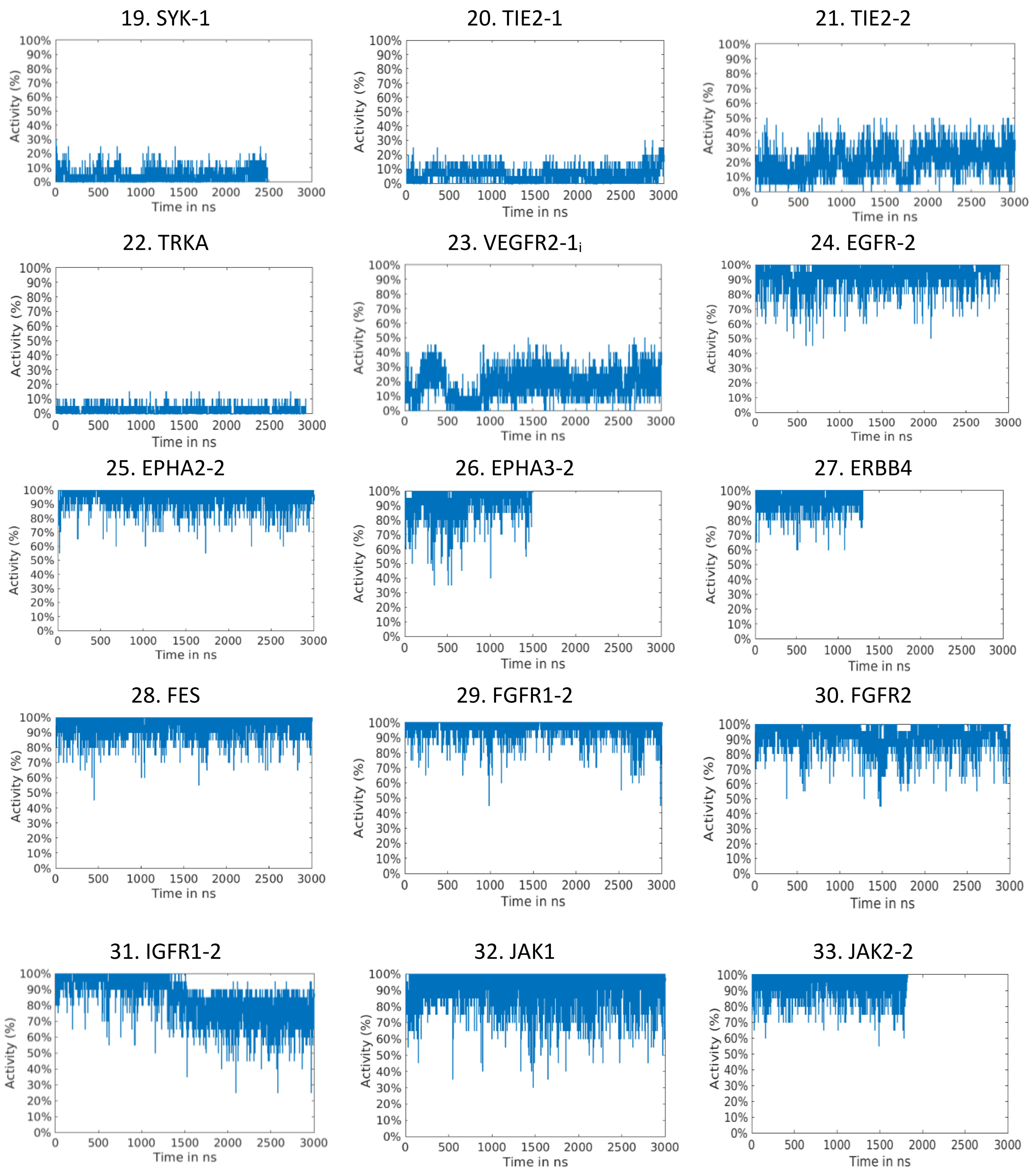
19. SYK-1
20. TIE2-1
21. TIE2-2
22. TRKA
23. VEGFR2-1$_i$
24. EGFR-2
25. EPHA2-2
26. EPHA3-2
27. ERBB4
28. FES
29. FGFR1-2
30. FGFR2
31. IGFR1-2
32. JAK1
33. JAK2-2
Activity (%)
Time in ns
0%
10%
20%
30%
40%
50%
60%
70%
80%
90%
100%
0
500
1000
1500
2000
2500
3000

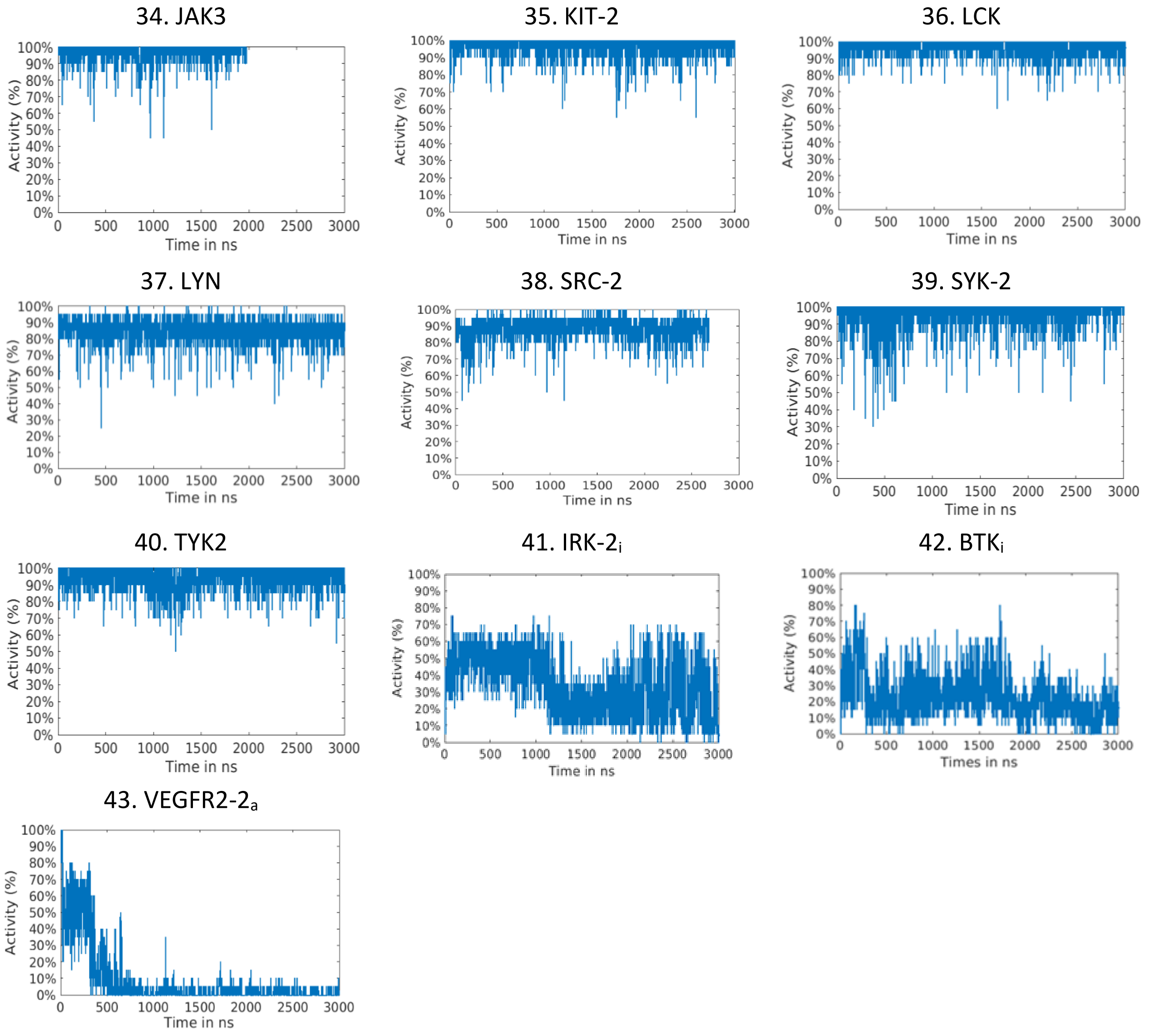

Figure S2. Estimated activity probability (using Kinconform[39,40]) of TK family proteins along simulation time.

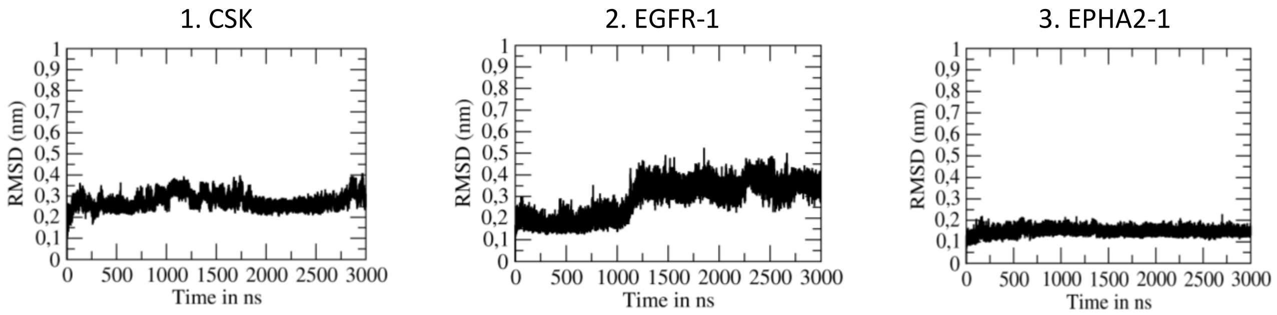

4. EPHA3-1

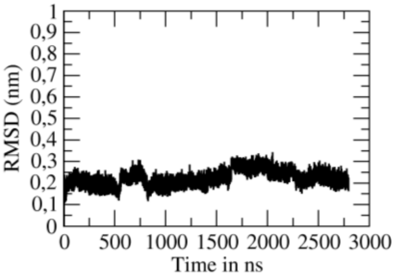

5. EPHB4

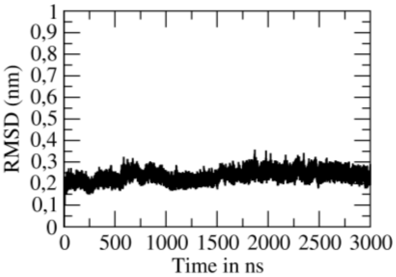

6. FGFR1-1

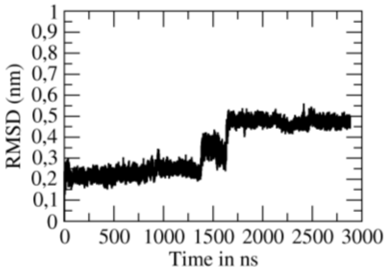

7. HCK

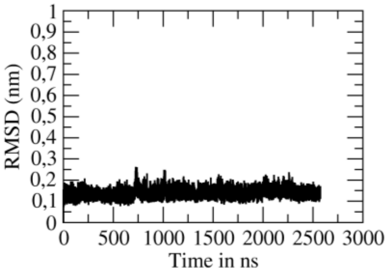

8. HER3

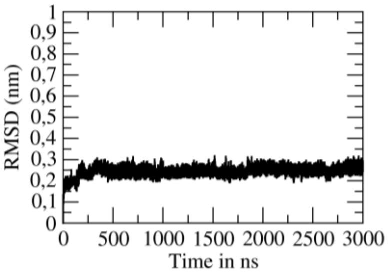

9. IGFR1-1

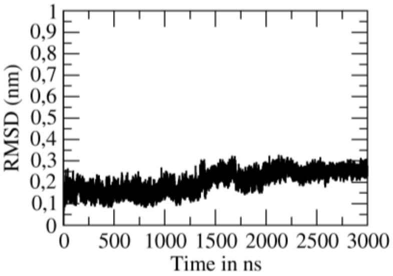

10. IRK-1$_i$

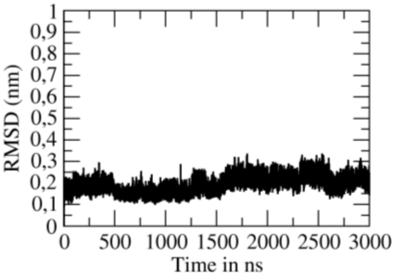

11. ITK

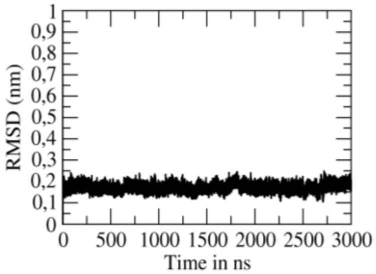

12. JAK2-1

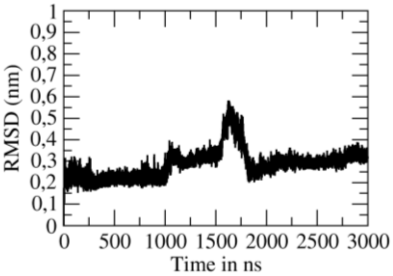

13. KIT-1

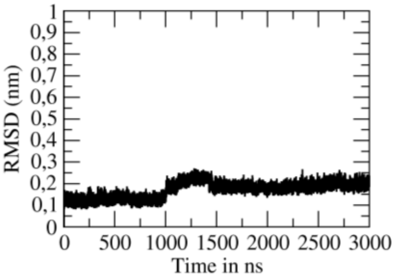

14. MET-1

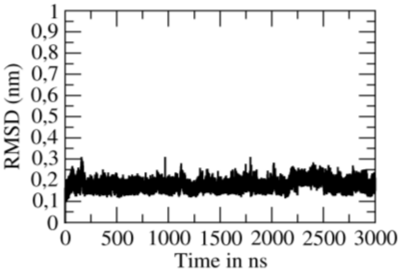

15. MET-2

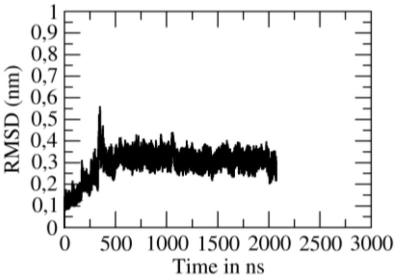

16. PYK2-1

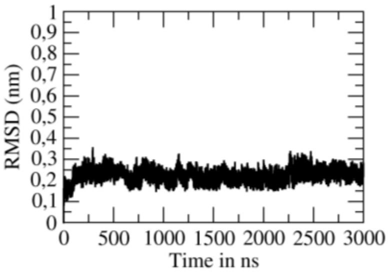

17. RON

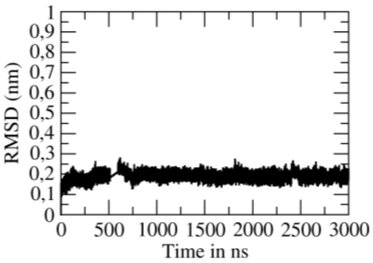

18. SRC-1

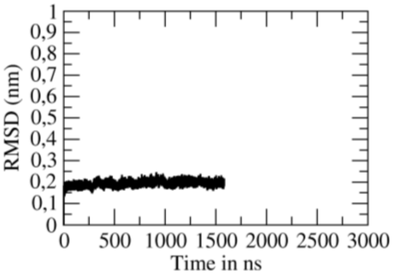

19. SYK-1

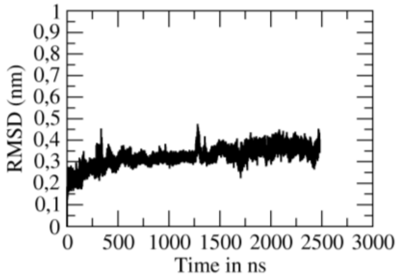

20. TIE2-1

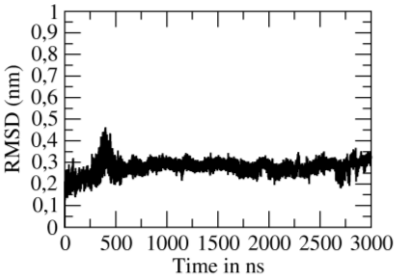

21. TIE2-2

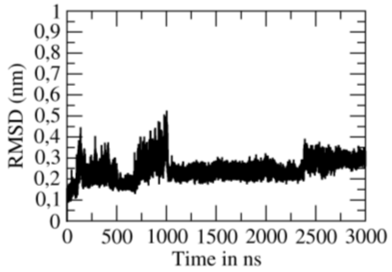

22. TRKA

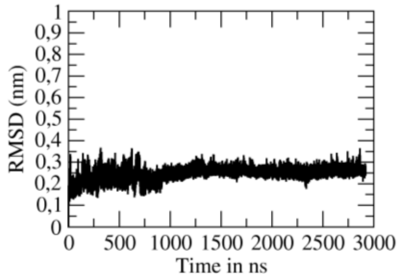

23. VEGFR2-1$_i$

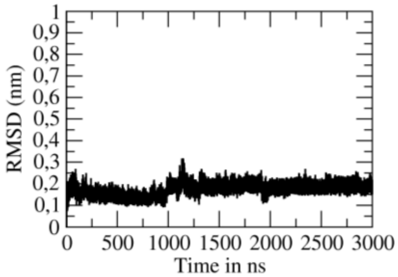

24. EGFR-2

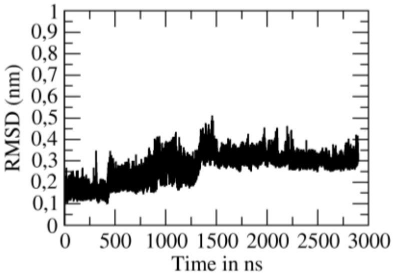

25. EPHA2-2

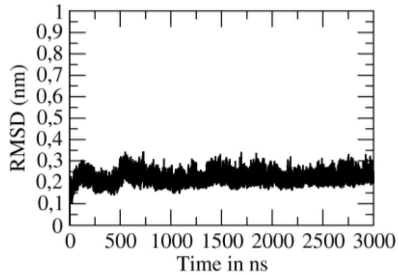

26. EPHA3-2

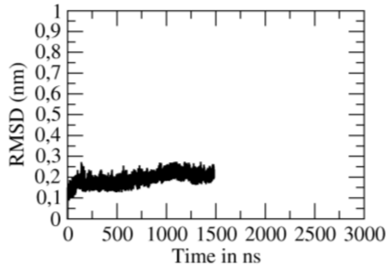

27. ERBB4

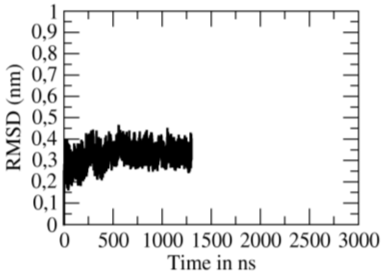

28. FES

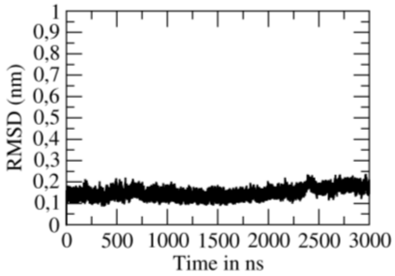

29. FGFR1-2

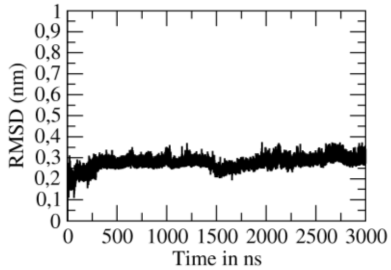

30. FGFR2

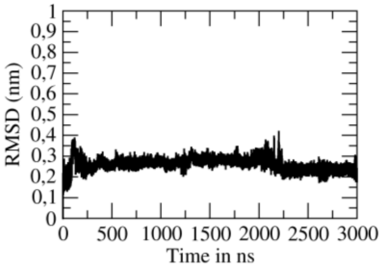

31. IGFR1-2

32. JAK1

33. JAK2-2

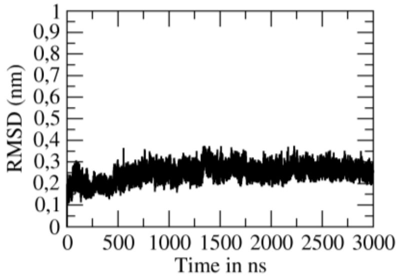

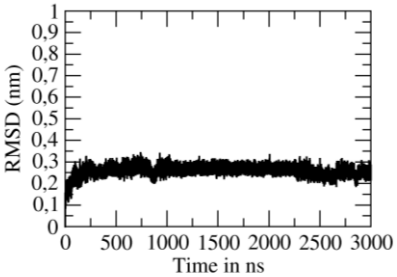

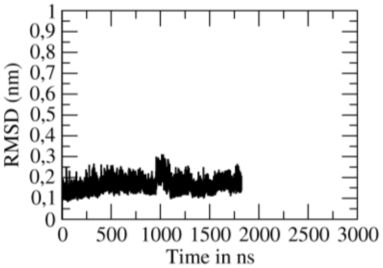

34. JAK3

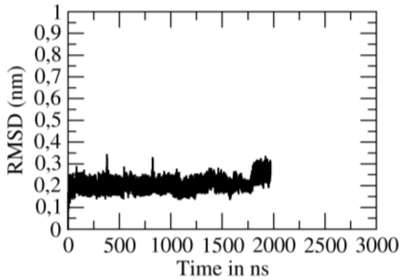

35. KIT-2

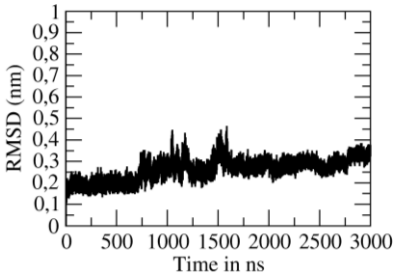

36. LCK

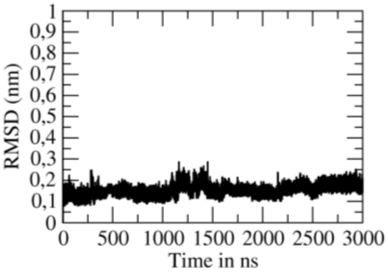

37. LYN

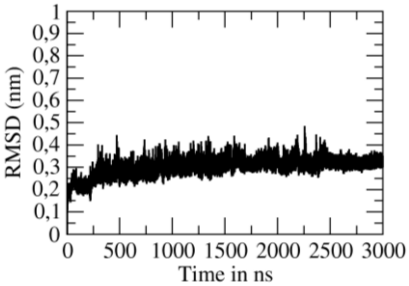

38. SRC-2

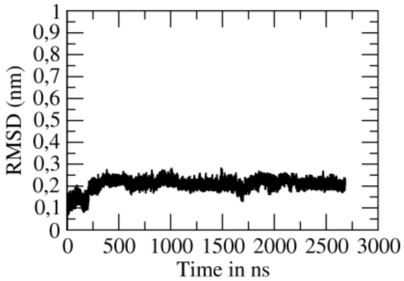

39. SYK-2

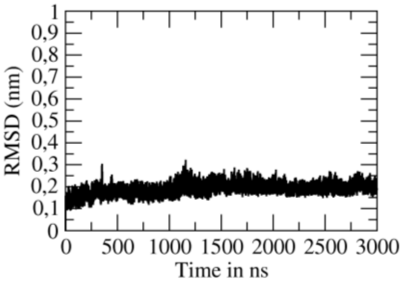

40. TYK2

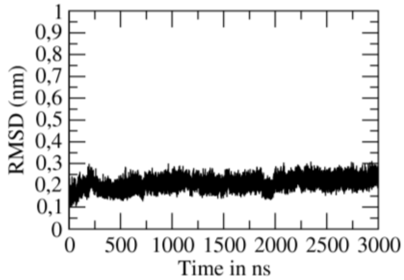

41. IRK-2_i

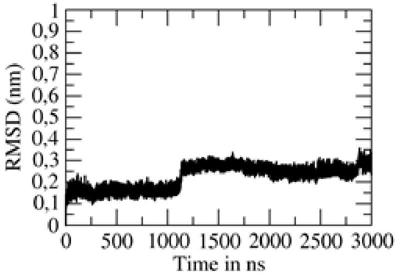

42. BTK_i

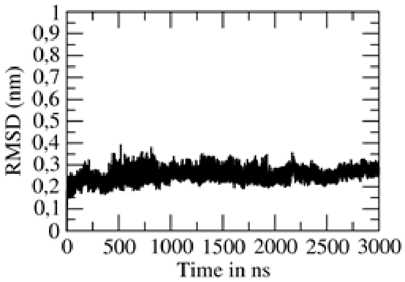

43. VEGFR2-2_a

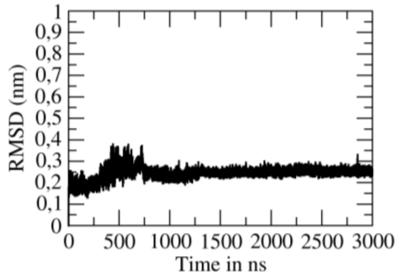

Figure S3. Root mean square deviation (RMSD) estimation of all the kinases considered for investigation.

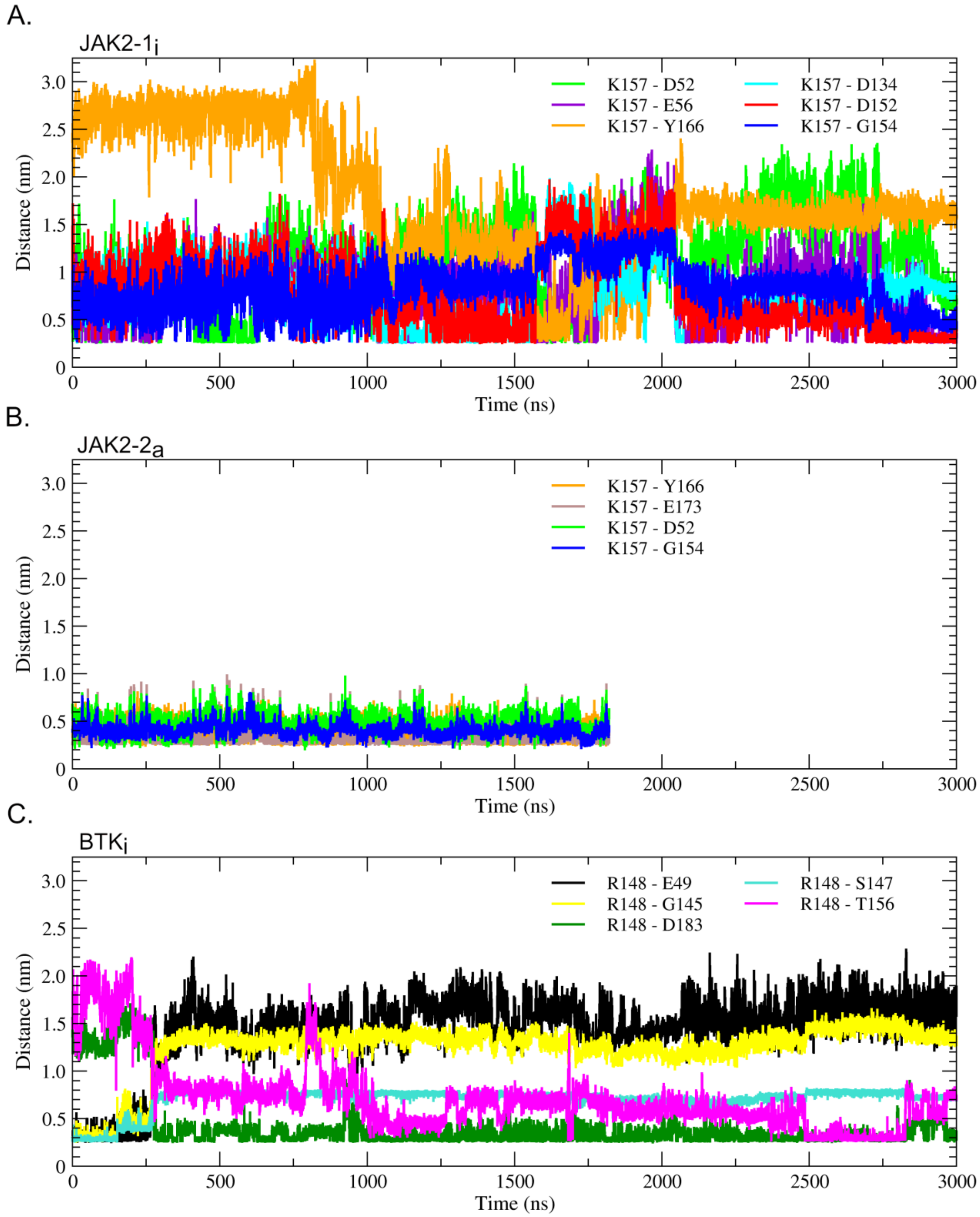

Figure S4. DFG+3 residue interactions along (**A**) JAK2-1$_i$ (PDB ID 3ugc, inactive)[41], (**B**) JAK2-2$_a$ (PDB ID 6bbv, active)[42] and (**C**) BTK$_i$ (PDB ID 1k2p, mixed behaviour)[36].

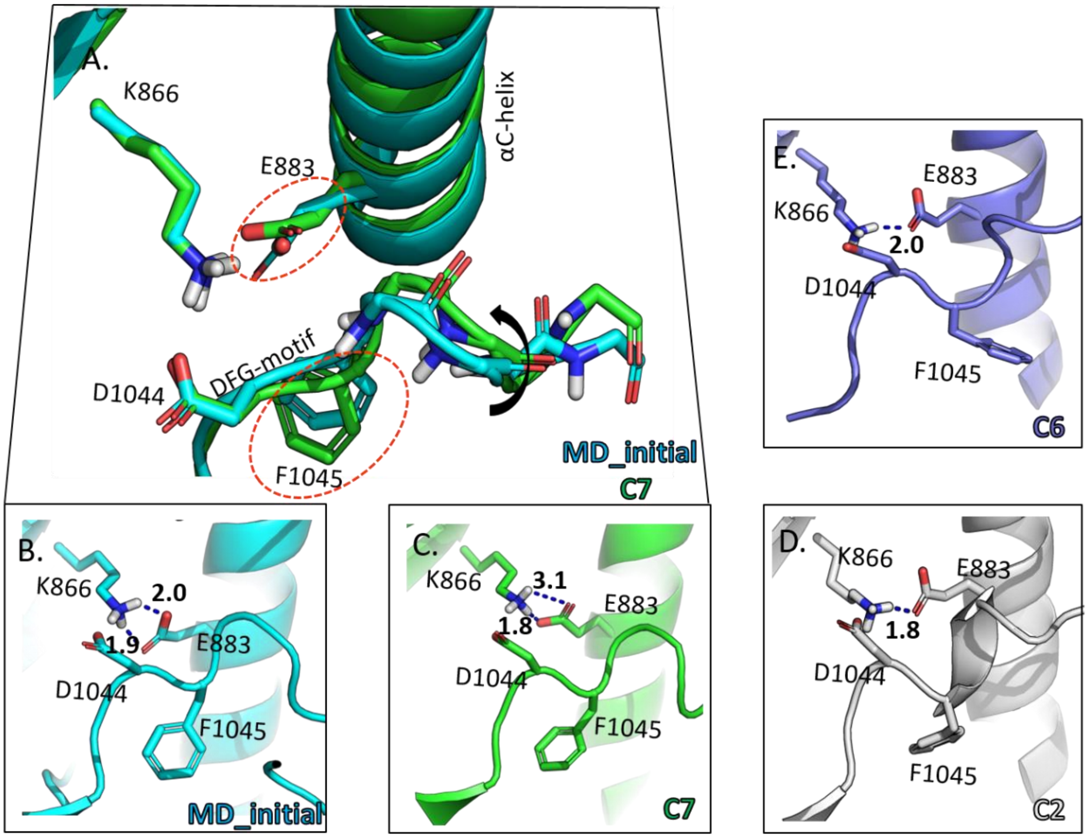

Figure S5. Structural comparison of several conformations of VEGFR2-2_a kinase via clustering analysis. **A**. Structural alignment of the initial frame from MD simulation (cyan) and C7 (green). For clarity, only the active site has been shown. The black arrow indicates a slightly closed 'active site/ATP-binding site' in C7 compared to MD initial frame. **B**. Conformation of the MD initial frame showing the K-E salt-bridge and DFG-motif. **C**. Conformation of C7 showing the K-E salt-bridge and DFG-motif. **D**. Conformation in C2 showing a weaker K-E salt-bridge. **E**. Conformation of C6 showing the weakest K-E salt-bridge configuration.

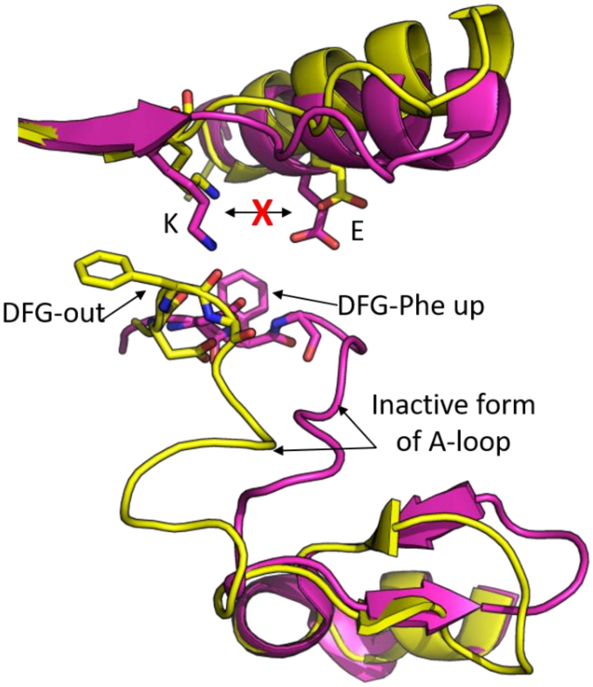

Figure S6. Structural comparison between the two inactive conformations: IRK-1_i (yellow, PDB ID 1irk[16]) and IRK-2_i (magenta, PDB ID 5hhw[35]).

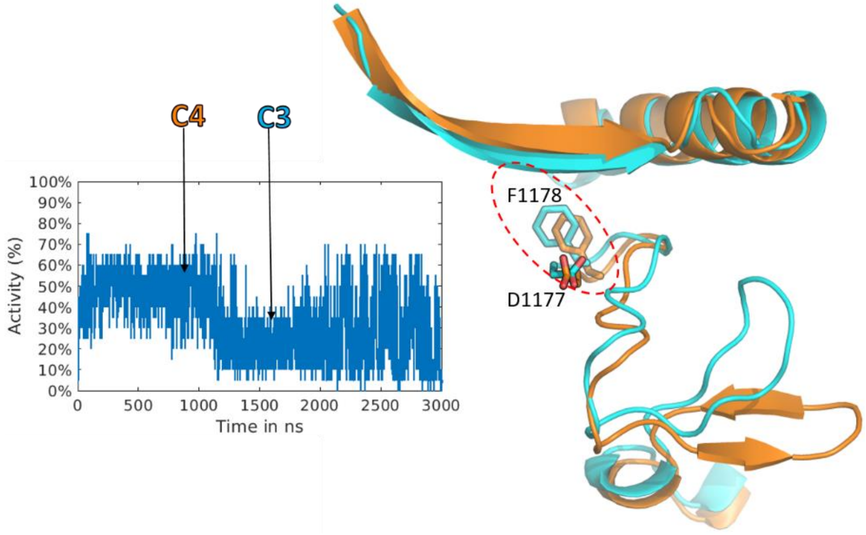

Figure S7. Conformations comparison for C4 and C3 of IRK-2_i. In both cases the "DFG-Phe" up conformation was observed. In C3, "DFG-Phe" was more frequently in the up conformation and blocking the active site of the kinase compared to C4.

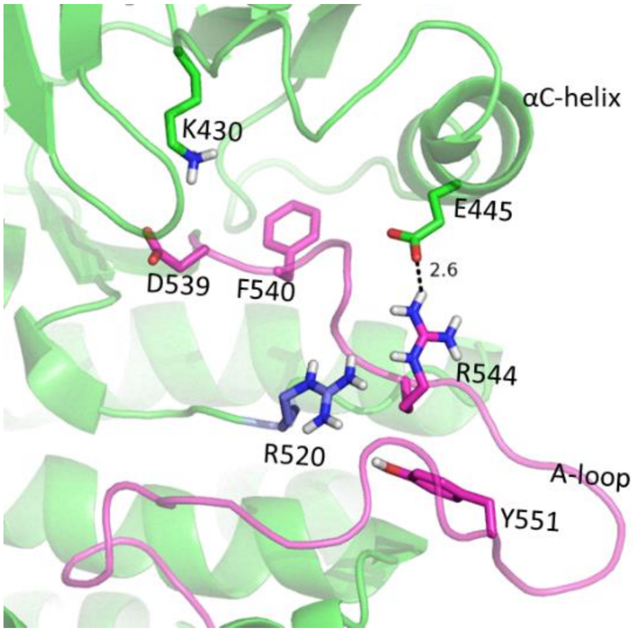

Figure S8. Inactive conformation of BTK$_i$ (PDB ID 1k2p[36]) as described by Chen et al[43]. Activation loop (A-loop) along with the DFG-motif has been shown in magenta. Residue R520 belongs to the HRD-motif, another conserved region, which is a part of the kinase catalytic loop. The αC-helix preserves the out conformation.

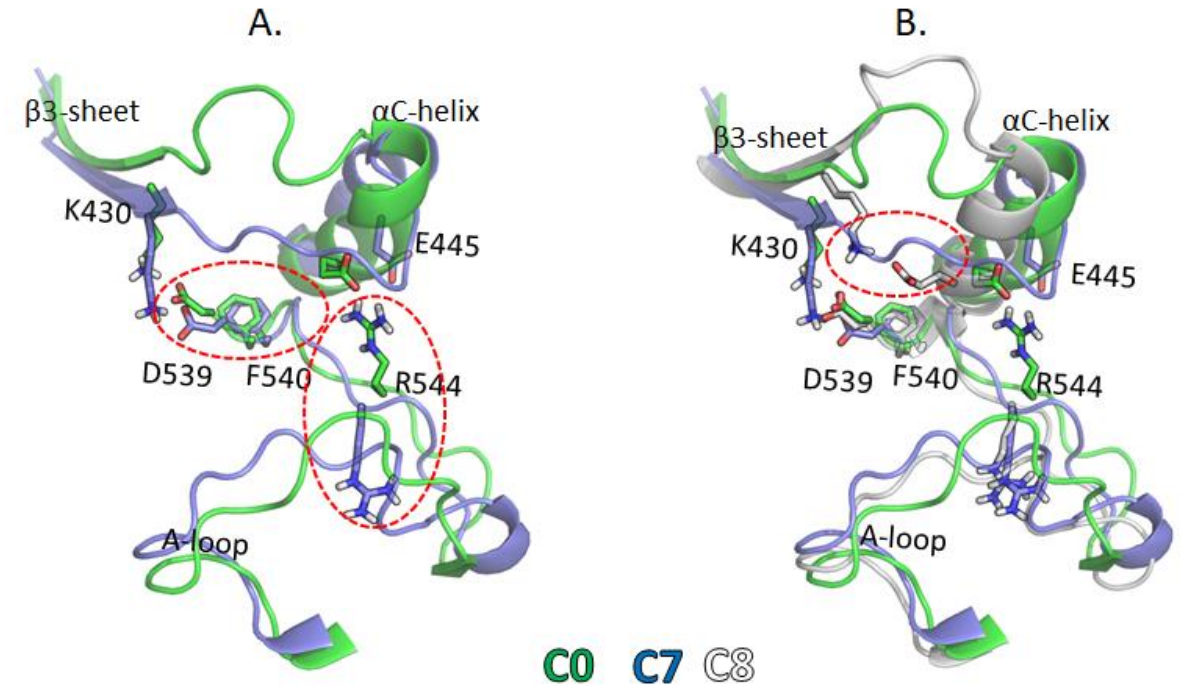

Figure S9. Major structural differences from the alignment of C0 (in green), C7 (in blue) and C8 (white) of BTK $_I$.

1. CSK

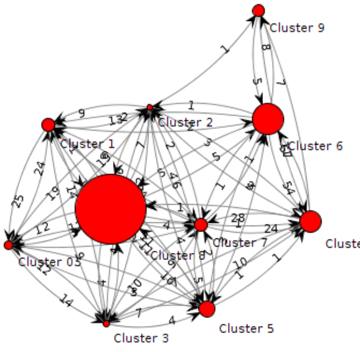

2. EGFR-1

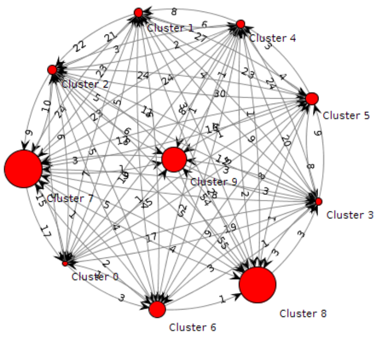

3. EPHA2-1

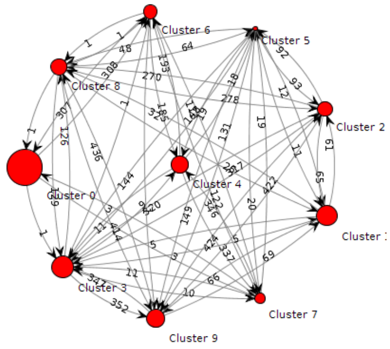

4. EPHA3-1

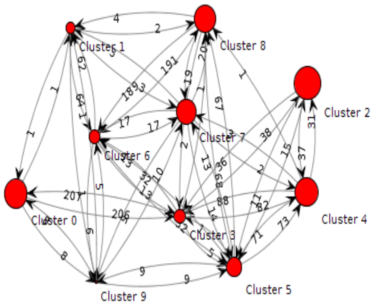

5. EPHB4

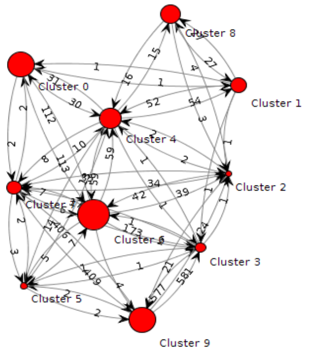

6. FGFR1-1

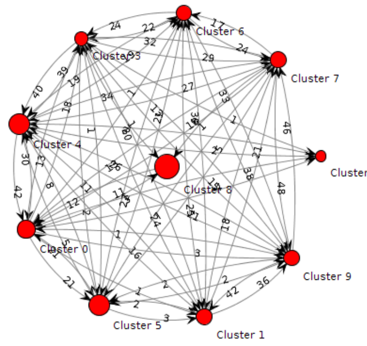

7. HCK

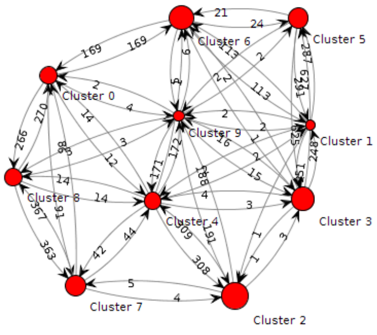

8. HER3

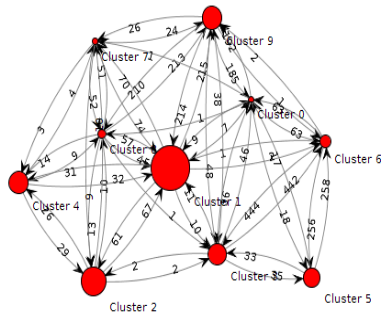

9. IGFR1-1

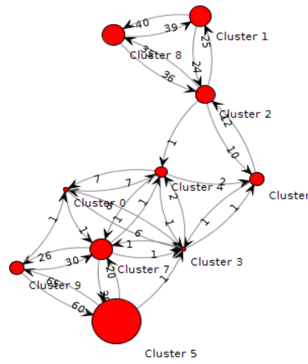

10. IRK-1$_i$

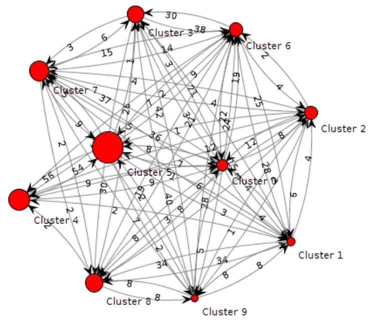

11. ITK

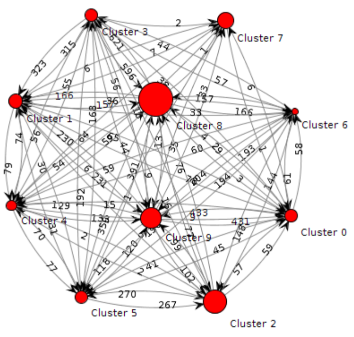

12. JAK2-1

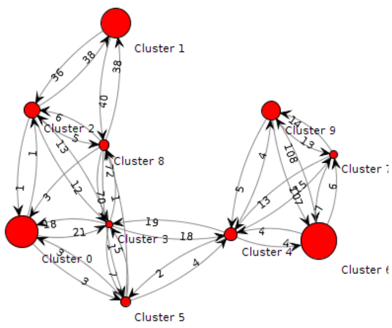

13. KIT-1

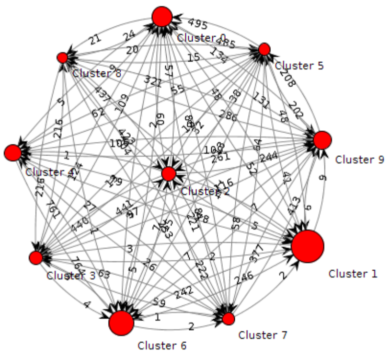

14. MET-1

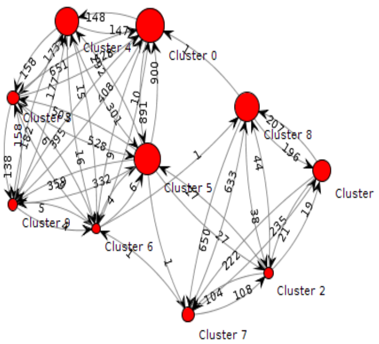

15. MET-2

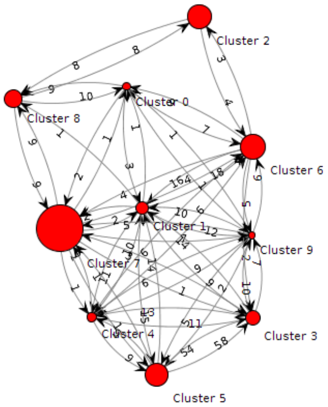

16. PYK2

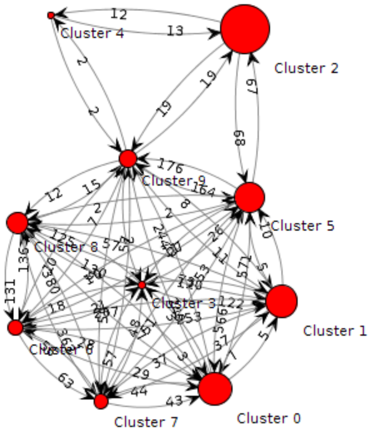

17. RON

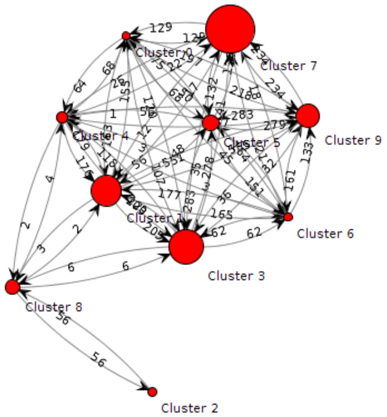

18. SRC-1

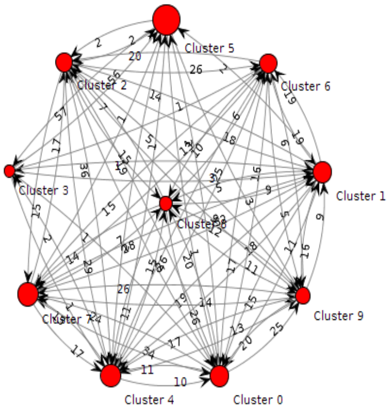

19. SYK-1

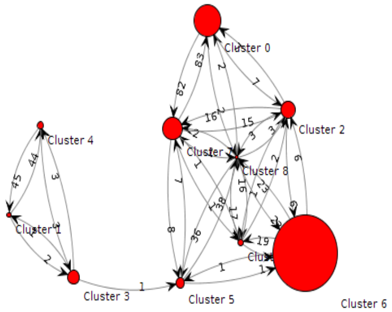

20. TIE2-1

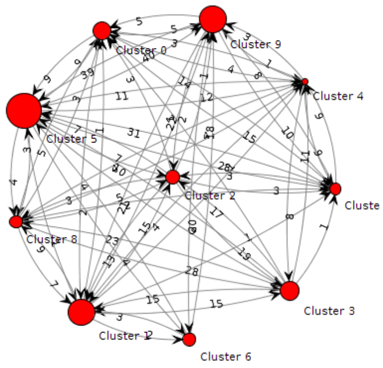

21. TIE2-2

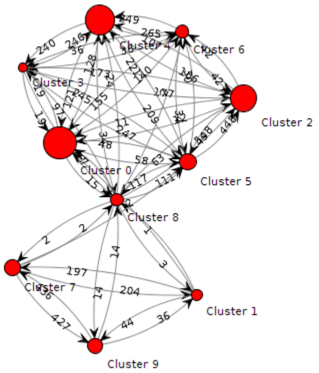

22. TRKA

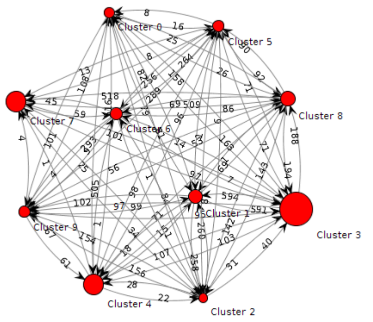

23. VEGFR2-1_i

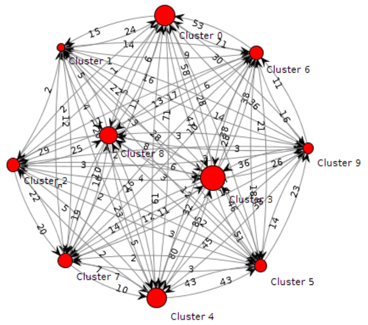

24. EGFR-2

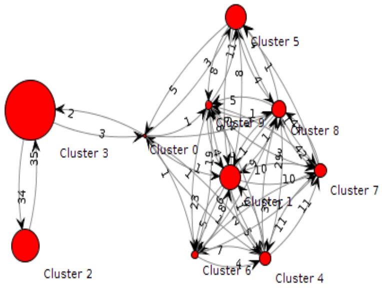

25. EPHA2-2

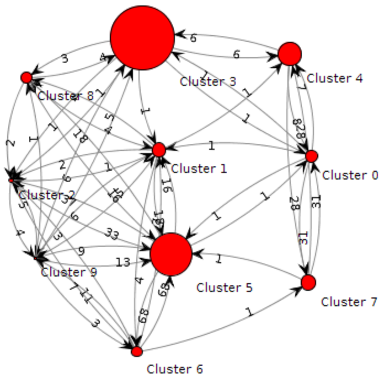

26. EPHA3-2

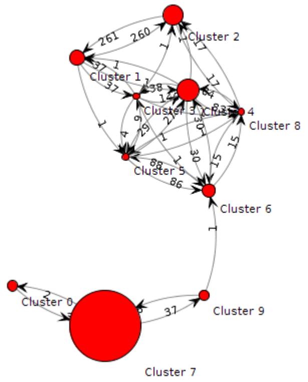

27. ERBB4

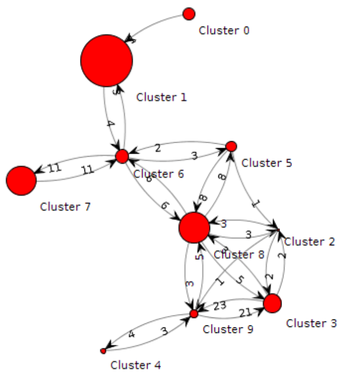

28. FES

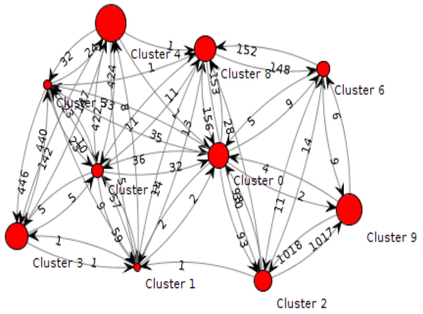

29. FGFR1-2

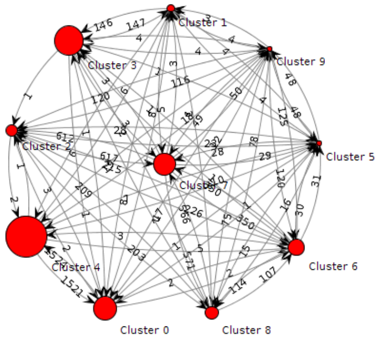

30. FGFR2

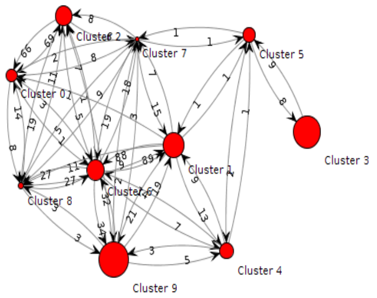

31. IGFR1-2

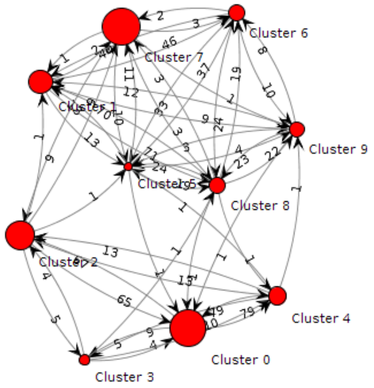

32. JAK1

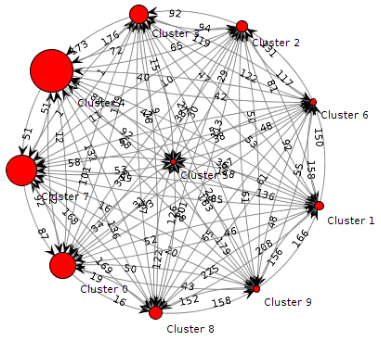

33. JAK2-2

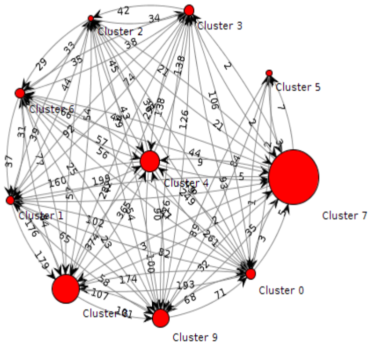

34. JAK3

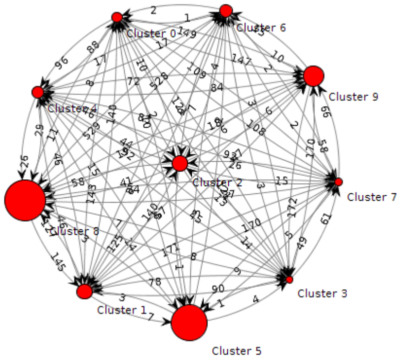

35. KIT-2

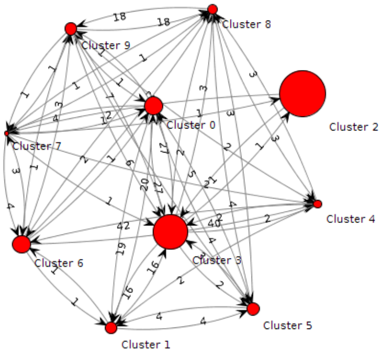

36. LCK

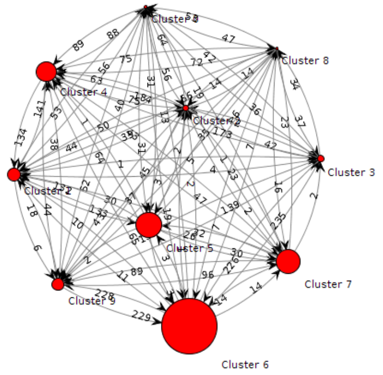

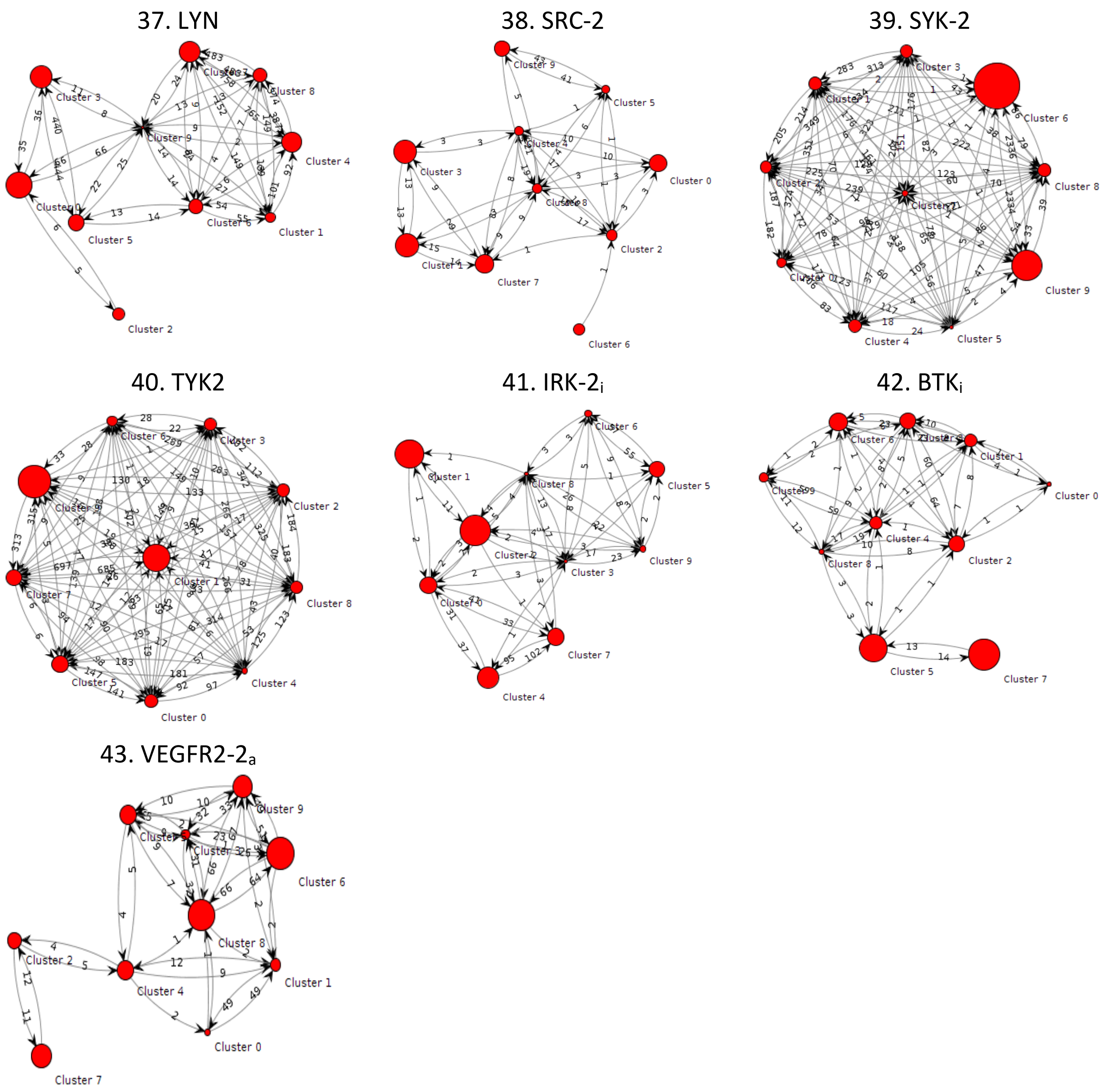

Figure S10. Clustering analysis of all the tyrosine kinases. We have used MD simulation trajectories to cluster conformations via the k-medoids algorithm[44] by considering all the markers used for activity classification. A fully connected graph denotes not significant conformational changes, whereas sparse graphs indicate more or less pronounced changes along time.

References

(1) Decherchi, S.; Bottegoni, G.; Spitaleri, A.; Rocchia, W.; Cavalli, A. BiKi Life Sciences: A New Suite for Molecular Dynamics and Related Methods in Drug Discovery. *J Chem Inf Model* **2018**, *58*, 219–224. https://doi.org/10.1021/ACS.JCIM.7B00680.

(2) Jorgensen, W. L.; Chandrasekhar, J.; Madura, J. D.; Impey, R. W.; Klein, M. L. Comparison of Simple Potential Functions for Simulating Liquid Water. *J Chem Phys* **1983**, *79*, 926–935. https://doi.org/10.1063/1.445869.

(3) Maier, J. A.; Martinez, C.; Kasavajhala, K.; Wickstrom, L.; Hauser, K. E.; Simmerling, C. Ff14SB: Improving the Accuracy of Protein Side Chain and Backbone Parameters from Ff99SB. **2015**. https://doi.org/10.1021/ACS.JCTC.5B00255.

(4) Abraham, M. J.; Murtola, T.; Schulz, R.; Páll, S.; Smith, J. C.; Hess, B.; Lindah, E. GROMACS: High Performance Molecular Simulations through Multi-Level Parallelism from Laptops to Supercomputers. *SoftwareX* **2015**, *1–2*, 19–25. https://doi.org/10.1016/J.SOFTX.2015.06.001.

(5) Páll, S.; Abraham, M. J.; Kutzner, C.; Hess, B.; Lindahl, E. Tackling Exascale Software Challenges in Molecular Dynamics Simulations with GROMACS. In *Lecture Notes in Computer Science (including subseries Lecture Notes in Artificial Intelligence and Lecture Notes in Bioinformatics)*; 2015; Vol. 8759. https://doi.org/10.1007/978-3-319-15976-8_1.

(6) Bussi, G.; Donadio, D.; Parrinello, M. Canonical Sampling through Velocity Rescaling. *J Chem Phys* **2007**, *126*, 14101. https://doi.org/10.1063/1.2408420.

(7) Parrinello, M.; Rahman, A. Polymorphic Transitions in Single Crystals: A New Molecular Dynamics Method. *J Appl Phys* **1998**, *52*, 7182. https://doi.org/10.1063/1.328693.

(8) Hess, B.; Bekker, H.; Berendsen, H. J. C.; Fraaije, J. G. E. M. LINCS: A Linear Constraint Solver for Molecular Simulations. *J Comput Chem* **1997**, *18*, 14631472. https://doi.org/10.1002/(SICI)1096-987X(199709)18:12.

(9) Darden, T.; York, D.; Pedersen, L. Particle Mesh Ewald: An N·log(N) Method for Ewald Sums in Large Systems. *J Chem Phys* **1998**, *98*, 10089. https://doi.org/10.1063/1.464397.

(10) Essmann, U.; Perera, L.; Berkowitz, M. L.; Darden, T.; Lee, H.; Pedersen, L. G. A Smooth Particle Mesh Ewald Method. *J Chem Phys* **1998**, *103*, 8577. https://doi.org/10.1063/1.470117.

(11) Lamers, M. B. A. C.; Antson, A. A.; Hubbard, R. E.; Scott, R. K.; Williams, D. H. Structure of the Protein Tyrosine Kinase Domain of C-Terminal Src Kinase (CSK) in Complex with Staurosporine. *J Mol Biol* **1999**, *285*, 713–725. https://doi.org/10.1006/JMBI.1998.2369.

(12) Yun, C. H.; Mengwasser, K. E.; Toms, A. v; Woo, M. S.; Greulich, H.; Wong, K. K.; Meyerson, M.; Eck, M. J. The T790M Mutation in EGFR Kinase Causes Drug Resistance by Increasing the Affinity for ATP. *Proceedings of the National Academy of Sciences* **2008**, *105*, 2070–2075. https://doi.org/10.1073/PNAS.0709662105.

(13) Dong, J.; Zhao, H.; Zhou, T.; Spiliotopoulos, D.; Rajendran, C.; Li, X. D.; Huang, D.; Caflisch, A. Structural Analysis of the Binding of Type I, I1/2, and II Inhibitors to Eph Tyrosine Kinases. *ACS Med Chem Lett* **2015**, *6*, 79–83. https://doi.org/10.1021/ML500355X.

(14) Tsai, J.; Lee, J. T.; Wang, W.; Zhang, J.; Cho, H.; Mamo, S.; Bremer, R.; Gillette, S.; Kong, J.; Haass, N. K.; Sproesser, K.; Li, L.; Smalley, K. S. M.; Fong, D.; Zhu, Y. L.; Marimuthu, A.; Nguyen, H.; Lam, B.; Liu, J.; Cheung, I.; Rice, J.; Suzuki, Y.; Luu, C.; Settachatgul, C.; Shellooe, R.; Cantwell, J.; Kim, S. H.; Schlessinger, J.; Zhang, K. Y. J.; West, B. L.; Powell, B.; Habets, G.; Zhang, C.; Ibrahim, P. N.; Hirth, P.; Artis, D. R.; Herlyn, M.; Bollag, G. Discovery of a Selective Inhibitor of Oncogenic B-Raf Kinase with Potent Antimelanoma Activity. *Proceedings of the National Academy of Sciences* **2008**, *105*, 3041–3046. https://doi.org/10.1073/PNAS.0711741105.

(15) Munshi, S.; Hall, D. L.; Kornienko, M.; Darke, P. L.; Kuo, L. C. Structure of Apo, Unactivated Insulin-like Growth Factor-1 Receptor Kinase at 1.5 Å Resolution. *Acta Crystallogr D Biol Crystallogr* **2003**, *59*, 1725–1730. https://doi.org/10.1107/S0907444903015415.

(16) Hubbard, S. R.; Wei, L.; Hendrickson, W. A. Crystal Structure of the Tyrosine Kinase Domain of the Human Insulin Receptor. *Nature* **1994**, *372*, 746–754. https://doi.org/10.1038/372746a0.

(17) Zapf, C. W.; Gerstenberger, B. S.; Xing, L.; Limburg, D. C.; Anderson, D. R.; Caspers, N.; Han, S.; Aulabaugh, A.; Kurumbail, R.; Shakya, S.; Li, X.; Spaulding, V.; Czerwinski, R. M.; Seth, N.; Medley, Q. G. Covalent Inhibitors of Interleukin-2 Inducible T Cell Kinase (ItK) with Nanomolar Potency in a Whole-Blood Assay. *J Med Chem* **2012**, *55*, 10047–10063. https://doi.org/10.1021/JM301190S.

(18) Gajiwaia, K. S.; Wu, J. C.; Christensen, J.; Deshmukh, G. D.; Diehl, W.; Dinitto, J. P.; English, J. M.; Greig, M. J.; He, Y. A.; Jacques, S. L.; Lunney, E. A.; McTigue, M.; Molina, D.; Quenzer, T.; Wells, P. A.; Yu, X.; Zhang, Y.; Zou, A.; Emmett, M. R.; Marshall, A. G.; Zhang, H. M.; Demetri, G. D. KIT Kinase Mutants Show Unique Mechanisms of Drug Resistance to Imatinib and Sunitinib in Gastrointestinal Stromal Tumor Patients. *Proceedings of the National Academy of Sciences* **2009**, *106*, 1542–1547. https://doi.org/10.1073/PNAS.0812413106.

(19) Xu, W.; Doshi, A.; Lei, M.; Eck, M. J.; Harrison, S. C. Crystal Structures of C-Src Reveal Features of Its Autoinhibitory Mechanism. *Mol Cell* **1999**, *3*, 629–638. https://doi.org/10.1016/S1097-2765(00)80356-1.

(20) Lovering, F.; McDonald, J.; Whitlock, G. A.; Glossop, P. A.; Phillips, C.; Bent, A.; Sabnis, Y.; Ryan, M.; Fitz, L.; Lee, J.; Chang, J. S.; Han, S.; Kurumbail, R.; Thorarensen, A. Identification of Type-II Inhibitors Using Kinase Structures. *Chem Biol Drug Des* **2012**, *80*, 657–664. https://doi.org/10.1111/J.1747-0285.2012.01443.X.

(21) Shewchuk, L. M.; Hassell, A. M.; Ellis, B.; Holmes, W. D.; Davis, R.; Horne, E. L.; Kadwell, S. H.; McKee, D. D.; Moore, J. T. Structure of the Tie2 RTK Domain: Self-Inhibition by the Nucleotide Binding Loop, Activation Loop, and C-Terminal Tail. *Structure* **2000**, *8*, 1105–1113. https://doi.org/10.1016/S0969-2126(00)00516-5.

(22) Stachel, S. J.; Sanders, J. M.; Henze, D. A.; Rudd, M. T.; Su, H. P.; Li, Y.; Nanda, K. K.; Egbertson, M. S.; Manley, P. J.; Jones, K. L. G.; Brnardic, E. J.; Green, A.; Grobler, J. A.; Hanney, B.; Leitl, M.; Lai, M. T.; Munshi, V.; Murphy, D.; Rickert, K.; Riley, D.; Krasowska-Zoladek, A.; Daley, C.; Zuck, P.;

Kane, S. A.; Bilodeau, M. T. Maximizing Diversity from a Kinase Screen: Identification of Novel and Selective Pan-Trk Inhibitors for Chronic Pain. *J Med Chem* **2014**, *57*, 5800–5816. https://doi.org/10.1021/JM5006429.

(23) Miyamoto, N.; Sakai, N.; Hirayama, T.; Miwa, K.; Oguro, Y.; Oki, H.; Okada, K.; Takagi, T.; Iwata, H.; Awazu, Y.; Yamasaki, S.; Takeuchi, T.; Miki, H.; Hori, A.; Imamura, S. Discovery of N-[5-({2-[(Cyclopropylcarbonyl)Amino]Imidazo[1,2-b]Pyridazin-6-Yl}oxy)-2-Methylphenyl]-1,3-Dimethyl-1H-Pyrazole-5-Carboxamide (TAK-593), a Highly Potent VEGFR2 Kinase Inhibitor. *Bioorg Med Chem* **2013**, *21*, 2333–2345. https://doi.org/10.1016/J.BMC.2013.01.074.

(24) Stamos, J.; Sliwkowski, M. X.; Eigenbrot, C. Structure of the Epidermal Growth Factor Receptor Kinase Domain Alone and in Complex with a 4-Anilinoquinazoline Inhibitor *. *Journal of Biological Chemistry* **2002**, *277*, 46265–46272. https://doi.org/10.1074/JBC.M207135200.

(25) Nowakowski, J.; Cronin, C. N.; McRee, D. E.; Knuth, M. W.; Nelson, C. G.; Pavletich, N. P.; Rogers, J.; Sang, B. C.; Scheibe, D. N.; Swanson, R. v; Thompson, D. A. Structures of the Cancer-Related Aurora-A, FAK, and EphA2 Protein Kinases from Nanovolume Crystallography. *Structure* **2002**, *10*, 1659–1667. https://doi.org/10.1016/S0969-2126(02)00907-3.

(26) Qiu, C.; Tarrant, M. K.; Choi, S. H.; Sathyamurthy, A.; Bose, R.; Banjade, S.; Pal, A.; Bornmann, W. G.; Lemmon, M. A.; Cole, P. A.; Leahy, D. J. Mechanism of Activation and Inhibition of the HER4/ErbB4 Kinase. *Structure* **2008**, *16*, 460–467. https://doi.org/10.1016/J.STR.2007.12.016.

(27) Filippakopoulos, P.; Kofler, M.; Hantschel, O.; Gish, G. D.; Grebien, F.; Salah, E.; Neudecker, P.; Kay, L. E.; Turk, B. E.; Superti-Furga, G.; Pawson, T.; Knapp, S. Structural Coupling of SH2-Kinase Domains Links Fes and Abl Substrate Recognition and Kinase Activation. *Cell* **2008**, *134*, 793–803. https://doi.org/10.1016/J.CELL.2008.07.047.

(28) Bae, J. H.; Lew, E. D.; Yuzawa, S.; Tomé, F.; Lax, I.; Schlessinger, J. The Selectivity of Receptor Tyrosine Kinase Signaling Is Controlled by a Secondary SH2 Domain Binding Site. *Cell* **2009**, *138*, 514–524. https://doi.org/10.1016/J.CELL.2009.05.028.

(29) Favelyukis, S.; Till, J. H.; Hubbard, S. R.; Miller, W. T. Structure and Autoregulation of the Insulin-like Growth Factor 1 Receptor Kinase. *Nature Structural Biology 2001 8:12* **2001**, *8*, 1058–1063. https://doi.org/10.1038/nsb721.

(30) Williams, N. K.; Bamert, R. S.; Patel, O.; Wang, C.; Walden, P. M.; Wilks, A. F.; Fantino, E.; Rossjohn, J.; Lucet, I. S. Dissecting Specificity in the Janus Kinases: The Structures of JAK-Specific Inhibitors Complexed to the JAK1 and JAK2 Protein Tyrosine Kinase Domains. *J Mol Biol* **2009**, *387*, 219–232. https://doi.org/10.1016/J.JMB.2009.01.041.

(31) Yamaguchi, H.; Hendrickson, W. A. Structural Basis for Activation of Human Lymphocyte Kinase Lck upon Tyrosine Phosphorylation. *Nature 1996 384:6608* **1996**, *384*, 484–489. https://doi.org/10.1038/384484a0.

(32) Cowan-Jacob, S. W.; Fendrich, G.; Manley, P. W.; Jahnke, W.; Fabbro, D.; Liebetanz, J.; Meyer, T. The Crystal Structure of a C-Src Complex in an Active Conformation Suggests Possible Steps in c-Src Activation. *Structure* **2005**, *13*, 861–871. https://doi.org/10.1016/J.STR.2005.03.012.

(33) Lam, B.; Arikawa, Y.; Cramlett, J.; Dong, Q.; de Jong, R.; Feher, V.; Grimshaw, C. E.; Farrell, P. J.; Hoffman, I. D.; Jennings, A.; Jones, B.; Matuszkiewicz, J.; Miura, J.; Miyake, H.; Natala, S. R.; Shi, L.; Takahashi, M.; Taylor, E.; Wyrick, C.; Yano, J.; Zalevsky, J.; Nie, Z. Discovery of TAK-659 an Orally Available Investigational Inhibitor of Spleen Tyrosine Kinase (SYK). *Bioorg Med Chem Lett* **2016**, *26*, 5947–5950. https://doi.org/10.1016/J.BMCL.2016.10.087.

(34) Chrencik, J. E.; Patny, A.; Leung, I. K.; Korniski, B.; Emmons, T. L.; Hall, T.; Weinberg, R. A.; Gormley, J. A.; Williams, J. M.; Day, J. E.; Hirsch, J. L.; Kiefer, J. R.; Leone, J. W.; Fischer, H. D.; Sommers, C. D.; Huang, H. C.; Jacobsen, E. J.; Tenbrink, R. E.; Tomasselli, A. G.; Benson, T. E. Structural and Thermodynamic Characterization of the TYK2 and JAK3 Kinase Domains in Complex with CP-690550 and CMP-6. *J Mol Biol* **2010**, *400*, 413–433. https://doi.org/10.1016/J.JMB.2010.05.020.

(35) Stauffer, F.; Cowan-Jacob, S. W.; Scheufler, C.; Furet, P. Identification of a 5-[3-Phenyl-(2-Cyclic-Ether)-Methylether]-4-Aminopyrrolo[2,3-d]Pyrimidine Series of IGF-1R Inhibitors. *Bioorg Med Chem Lett* **2016**, *26*, 2065–2067. https://doi.org/10.1016/J.BMCL.2016.02.074.

(36) Mao, C.; Zhou, M.; Uckun, F. M. Crystal Structure of Bruton's Tyrosine Kinase Domain Suggests a Novel Pathway for Activation and Provides Insights into the Molecular Basis of X-Linked Agammaglobulinemia *. *Journal of Biological Chemistry* **2001**, *276*, 41435–41443. https://doi.org/10.1074/JBC.M104828200.

(37) la Sala, G.; Decherchi, S.; de Vivo, M.; Rocchia, W. Allosteric Communication Networks in Proteins Revealed through Pocket Crosstalk Analysis. *ACS Cent Sci* **2017**, *3*, 949–960. https://doi.org/10.1021/ACSCENTSCI.7B00211.

(38) Crooks, G. E.; Hon, G.; Chandonia, J.-M.; Brenner, S. E. WebLogo: A Sequence Logo Generator. **2004**, 1188–1190. https://doi.org/10.1101/gr.849004.

(39) McSkimming, D. I.; Rasheed, K.; Kannan, N. Classifying Kinase Conformations Using a Machine Learning Approach. *BMC Bioinformatics* **2017**, *18*. https://doi.org/10.1186/S12859-017-1506-2.

(40) McSkimming, D. I. Esbg/Kinconform: Kinconform. 2017. https://doi.org/10.5281/zenodo.249090.

(41) Andraos, R.; Qian, Z.; Bonenfant, D.; Rubert, J.; Vangrevelinghe, E.; Scheufler, C.; Marque, F.; Régnier, C. H.; de Pover, A.; Ryckelynck, H.; Bhagwat, N.; Koppikar, P.; Goel, A.; Wyder, L.; Tavares, G.; Baffert, F.; Pissot-Soldermann, C.; Manley, P. W.; Gaul, C.; Voshol, H.; Levine, R. L.; Sellers, W. R.; Hofmann, F.; Radimerski, T. Modulation of Activation-Loop Phosphorylation by JAK Inhibitors Is Binding Mode Dependent. *Cancer Discov* **2012**, *2*, 512–523. https://doi.org/10.1158/2159-8290.CD-11-0324.

(42) Vazquez, M. L.; Kaila, N.; Strohbach, J. W.; Trzupek, J. D.; Brown, M. F.; Flanagan, M. E.; Mitton-Fry, M. J.; Johnson, T. A.; Tenbrink, R. E.; Arnold, E. P.; Basak, A.; Heasley, S. E.; Kwon, S.; Langille, J.; Parikh, M. D.; Griffin, S. H.; Casavant, J. M.; Duclos, B. A.; Fenwick, A. E.; Harris, T. M.; Han, S.; Caspers, N.; Dowty, M. E.; Yang, X.; Banker, M. E.; Hegen, M.; Symanowicz, P. T.; Li, L.; Wang, L.; Lin, T. H.; Jussif, J.; Clark, J. D.; Telliez, J. B.; Robinson, R. P.; Unwalla, R. Identification of N-{cis-3-[Methyl(7H-Pyrrolo[2,3-d]Pyrimidin-4-Yl)Amino]Cyclobutyl}propane-1-Sulfonamide (PF-

04965842): A Selective JAK1 Clinical Candidate for the Treatment of Autoimmune Diseases. *J Med Chem* **2018**, *61*, 1130–1152. https://doi.org/10.1021/ACS.JMEDCHEM.7B01598.

(43) Chen, X.; Leyendecker, S.; Bedem, H. van den. Kinematic Flexibility Analysis of Active and Inactive Kinase Conformations. *PAMM* **2021**, *20*, e202000166. https://doi.org/10.1002/PAMM.202000166.

(44) Park, H. S.; Jun, C. H. A Simple and Fast Algorithm for K-Medoids Clustering. *Expert Syst Appl* **2009**, *36*, 3336–3341. https://doi.org/10.1016/J.ESWA.2008.01.039.
